# Nature’s Green Potential: Anticancer Properties of Plants of the Euphorbiaceae Family

**DOI:** 10.3390/cancers16010114

**Published:** 2023-12-25

**Authors:** Víctor Jiménez-González, Tomasz Kowalczyk, Janusz Piekarski, Janusz Szemraj, Patricia Rijo, Przemysław Sitarek

**Affiliations:** 1 Department of Pharmacology, Faculty of Pharmacy, University of Seville, 41012 Seville, Spain; 2 Department of Molecular Biotechnology and Genetics, Faculty of Biology and Environmental Protection, University of Lodz, Banacha 12/16, 90-237 Lodz, Poland; 3 Department of Surgical Oncology, Medical University in Lodz, 93-513 Lodz, Poland; janusz.piekarski@umed.lodz.pl; 4 Department of Medical Biochemistry, Medical University of Lodz, 92-215 Lodz, Poland; janusz.szemraj@umed.lodz.pl; 5 CBIOS-Lusófona University’s Research Center for Biosciences and Health Technologies, 1749-024 Lisbon, Portugal; p1609@ulusofona.pt; 6 Instituto de Investigação do Medicamento (iMed.ULisboa), Faculdade de Farmácia, Universidade de Lisboa, 1649-003 Lisbon, Portugal; 7 Department of Medical Biology, Medical University of Lodz, 90-151 Lodz, Poland; przemyslaw.sitarek@umed.lodz.pl

**Keywords:** Euphorbiaceae family, in vitro, in vivo, anticancer, extracts, pure compounds, nanoparticles

## Abstract

**Simple Summary:**

Euphorbiaceae is a large family of flowering plants that includes a wide spectrum of useful plants, from edible plants to toxic and medicinal plants. They are cosmopolitan plants that have very different shapes, from little herbaceous plants to big trees and cactus-like forms. This review article focuses on the potential anticancer activity of extracts, isolated compounds, and nanoparticles generated from the plants of the Euphobiaceae family based on in vitro and in vivo experiments. Possible mechanisms of action are also discussed.

**Abstract:**

The number of cancer cases will reach 24 million in 2040, according to the International Agency for Research on Cancer. Current treatments for cancer are not effective and selective for most patients; for this reason, new anticancer drugs need to be developed and researched enough. There are potentially useful drugs for cancer isolated from plants that are being used in the clinic. Available information about phytochemistry, traditional uses, in vitro and in vivo experiments with plants, and pure compounds isolated from the Euphorbiaceae family indicates that this family of plants has the potential to develop anticancer drugs. This review examines selected species from the Euphorbiaceae family and their bioactive compounds that could have potential against different types of cancer cells. It reviews the activity of crude extracts, isolated compounds, and nanoparticles and the potential underlying mechanisms of action.

## 1. Introduction

Nowadays, cancer is a serious health problem that represents a great cost to national health systems around the world. Usually cells live, repair errors, divide, and die, and new cells replace the old ones. But sometimes, a mutation in the DNA of a cell that can not repair itself through reparation mechanisms leads to multiple abnormal divisions. These multiple divisions could activate oncogenes (inducing cell growth) or/and deactivate tumor suppressor genes (repressing cell growth), leading to an uncontrolled cell cycle. All these fast divisions, without control, accumulate different mutations that could imply fast growth and cancer cell death evasion. These mutations could also favor the loss of adhesion of tumor cells, which would facilitate their movement to other areas of the body through epithelial-mesenchymal transition, promoting tumor invasion and metastasis. In addition, it has been established that tumors release some angiogenic cytokines that affect vessel formation, tumor development, invasion, and metastasis. The most important angiogenic cytokines are vascular endothelial growth factor (VEGF) and basic fibroblast growth factor (bFGF), which are also poor markers of the prognostic and aggressiveness of the illness in patients [1,2].

More than 19 million new cases of cancer and 10 million deaths from cancer were reported in 2020 [3]. The most frequently diagnosed cancer was breast cancer (11.7%), lung cancer (11.6%), colorectal cancer (10%), and prostate cancer (7.3%). Furthermore, estimates made by experts expect that in 2040 there will be 24.8 million cases of cancer (47% more than in 2020) [4]. With this future perspective, new treatments and the development of new drugs will be difficult to achieve if governments do not increase the share devoted to cancer, and this lack of financing may be a problem in receiving quality care [5]. New therapies for cancer are usually directed toward developing more effective drugs and trying to avoid the side effects of the actual anticancer drugs. But all these strategies are usually very complex or hard and expensive to achieve, using new molecular targets, nanomedicine, and bioengineering [6]. However, the plant kingdom is still being unexplored; nonetheless, several very useful anticancer drugs are of plant origin, such as Paclitaxel, Vincristine, Vinblastine, Irinotecan, Topotecan, and Etoposide [7,8,9].

The Euphorbiaceae family of plants is a group of plants that has gained the interest of the scientific community due to their traditional uses in ethnomedicine, their high diversity of compounds, the potential toxicity of these compounds, and their easy access to these cosmopolitan plants [10]. Different plants from the genus of the family, such as Euphorbia, Croton, Jatropha, and Cnidoscolus, have been tested in vitro for their anticancer activity with excellent results [11,12,13]. This gives us an opportunity to continue delving deeper into the compounds present in these plants and their mechanisms of action as potential anticancer drugs. Several secondary metabolites have been described in the Euphorbiaceae family; for example, there is a high diversity of terpenoids with different types of original skeleton configurations [14,15,16]. Some common flavonoids, like quercetin and apigenin, have been isolated from this plant family [17,18,19,20]. Additionally, it has been documented that these plants contain certain tryptamine-derived alkaloids [21].

The main purpose of this review is to bring together all the information available about the anticancer effect in vitro or in vivo of plant extracts or pure compounds isolated from the Euphorbiaceae family published in the last ten years.

## 2. Criteria for the Selection of Experimental Papers

This review is based on primary literature published between 2012 and 2023 (to date). The papers were selected from different electronic databases: PubMed, Google Scholar, Scopus, and Web of Science. The following terms were used to achieve the search: Euphorbiaceae with: family, anticancer, antiproliferative, cytotoxic, plant extract, pure compound, in vitro, in vivo, and nanoparticles. The articles reporting extracts or pure compounds from the Euphorbiaceae family with some anticancer, antiproliferative, or cytotoxic activity in vitro or in vivo were included. Other articles reporting different types of reviews, articles in different languages than English, articles without full text access, lacking specific plant names, without reports of clear objectives and methodology, published more than ten years ago, using plant species other than Euphorbiaceae, were excluded. Duplicate articles from different database searching results were also excluded. All the inclusion/exclusion criteria were checked again after the removal of these articles. Each selected research paper was examined, and the following data were selected and presented in the tables: scientific plant name, parts of the plants used for extract preparation or pure compound isolation, type of extract, class of compounds or different compounds identified in the extract, cancer cell lines used or animal model/cell line inoculated with cancer-inducing compounds, activity or mechanism of action, and reference. Articles explaining the mechanisms of action of Euphorbiaceae plant extracts or isolated compounds were discussed before the tables in the main text. 

## 3. The Euphorbiaceae Family of Plants

**Figure 1:**
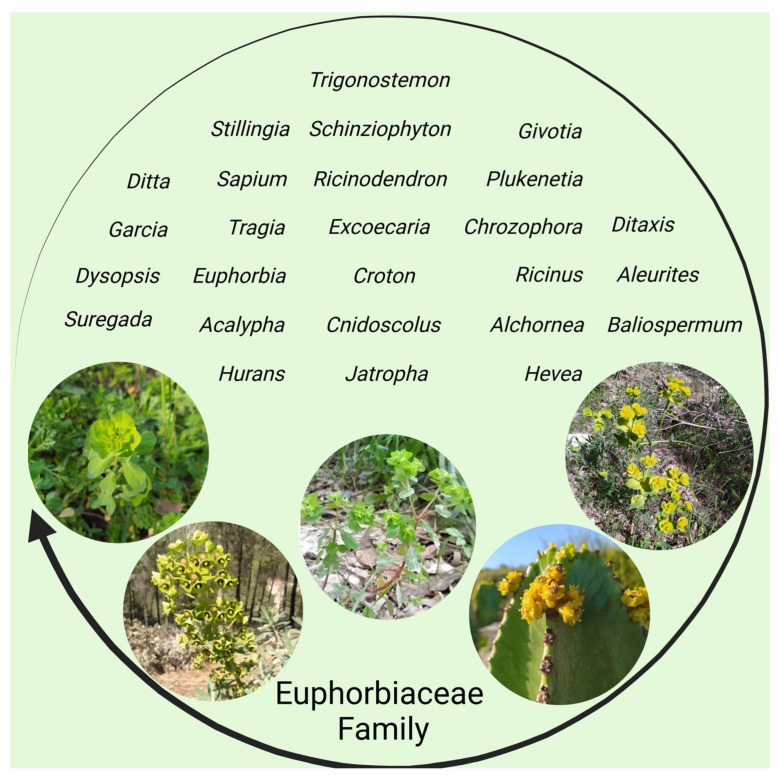
The Euphorbiaceae family of green plants. (created by BioRender).

The Euphorbiaceae family of plants has about 228 plant genera accepted according to Plants of the World Online by Royal Botanic Garden Kew [22] and more than 7000 species of plants according to the Global Biodiversity Information Facility [23]. This family of green plants has a very different overall shape. There are habits from herbaceous plants (for example, *Euphorbia peplus* L.) to shrubs (like *Ricinus communis* L.) or woody trees (*Hevea brasiliensis* (Willd. ex A.Juss.) Müll.Arg.) and cactus-like shapes (*Euphorbia ingens* (E.Mey. ex Boiss.)). These plants can be annuals or perennials, monoics or dioics, and usually present some type of latex. The leaves are oppositive (sometimes alternatives) and have stipules that could be transformed into spines or glands. The flowers gather in an inflorescence called cyathium, and sometimes they are inconspicuous flowers that lack a corolla. Sometimes the cyathium contains several masculine flowers and only one feminine flower. Androecium with one or numerous stamens, and gynoecium with two or three styles and carpels. The fruits are usually contained in capsules, and they separate easily from each other [24,25,26]. Among the best-known genera of this family, we find *Euphorbia*, *Jatropha*, *Croton*, *Acalypha*, *Cnidoscolus*, and *Ricinus* (Figure 1) [22]. Some plants in the family are very important for humans; for example, *Mannihot sculenta* L. is an edible plant that is cultivated in many countries and is considered a basic food. This crop is easy to grow in a variety of environments, is undemanding, and has very good nutritional properties [27,28]. *Mannihot sculenta* L. was grown as a crop on more than one million hectares in countries such as Ghana, Angola, Cote d’Ivoire, Brazil, Thailand, the Democratic Republic of the Congo, and Nigeria in 2021, according to the Food and Agriculture Organization of the United Nations [29]. Other plants are considered toxic to be consumed but still useful; for example, in the proper dosage, *Ricinus communis* L., commonly known as Castor oil, has been used in the past as a purgative [30] and is approved by the US Food and Drug Administration as an antisticking additive agent to produce hard candy [31]. Many other uses of castor’s oil have been reported, including uterine contraction [32], antiviral [33], antibacterial [34], and antinflammatory [30]. In addition, the oils extracted from the seeds are very useful for industrial applications [31,35].

Plants of the most numerous genera, *Euphorbia*, have been used traditionally around the world for different purposes, for example, to feed animals, as additives in food, as fuels, or for environmental uses, but the two main purposes have been to poison different animals and as folk medicines [10]. These two main purposes take advantage of the toxicological properties of the diverse phytochemistry present in these plants. For example, it is well known that different species of Euphorbiaceae, for example, *Euphorbia tirucalli* L., have been used in Africa to poison fish. This is a very common way of fishing with the help of plant metabolites that spread through water and poison animals [36]. On the other hand, when used as folk medicines, they are known to be used mainly for digestive disorders, skin or subcutaneous tissue disorders, infections, inflammation, or respiratory disorders [10]. For example, for digestive disorders, decoctions of *Euhprobia hirta* L. are recorded to be used in distant places such as Burundi, the Philippines, and China as antidiarrheal [37,38,39]. *Euphorbia lathyris* L. is recorded as being used as a purgative in different countries in Europe. For skin disorders, *Euphorbia maculata* L. is well known to be used in folk medicine against warts [40]. To treat infections, for example, *Euphorbia hirta* L. had been used in the past for gonorrhoea in Africa [41]. The same plant has been reported to be used in Australia for bronchitis (inflammation) [42]. For respiratory disorders, it is used in Nepal for the treatment of asthma [43].

### 3.1. Phytochemistry of the Euphorbiaceae Family

This family of plants is very diverse and rich in secondary metabolites. Different compounds have been isolated, for example, terpenoids and flavonoids. The first class of compounds in Euphorbiaceae are terpenoids, classified into diterpenoids, triterpenoids, and sesquiterpenoids. These metabolites have been studied in this family for a long time for their different biological activities. The diterpenoids are cyclized from the precursor geranyl-geranyl-phosphate to the corresponding diterpenoid skeleton. In the Euphorbiaceae family, compounds have been isolated with different types of diterpenoid skeletons [44,45,46].

For example, in the genus *Euphorbia*, different types of diterpenoids have been described, such as labdane, abietane, atisane, kaurane, isopimarane, rosane, dolabrane, casbane, cembrane, rharnnofolane, gaditanone, ingenane, ingol, jatrophane, jatropholane, lathyrane, cyclomyrsinol, myrsinol, premyrsinol, paraliane, pepluane, presegetane, segetane, tigliane, cyclojatrophane, and expoxyjatropholane [44,45]. Due to the high diversity of plants and metabolites in this genus, every year new diterpenoids are discovered. Zhu et al. [47] using the roots *Euphorbia fischeriana* L. described two new *ent*-abietane diterpenoids, euphonoids H and I. Zhang et al. [48] using the same plant species have reported for the first time the presence of a new *ent*-rosane diterpene, named ebracteolatas D. On the other hand, in the *Croton* genus, labdanes, clerodanes, trachylobane, kaurene, cembrane, and isopimarane diterpenoids have been identified [46]. Wang et al. [49] reported the isolation of a new *ent*-abietane diterpenoid (7b,13a,15-tri-hydroxy-*ent*-abieta-8(14)-en-3-one) from the leaves of *Croton lachnocarpus* Benth. In the genus *Jatropha*, different diterpenoids’ skeletons have also been described, such as tigliane, casbene, daphnane, lathyrane, jatrophane, podocarpane, and rhamnofolane [50]. For example, the team led by Yuan et al. [51] isolated jatropodagins A, a new lathyrane-type diterpenoid. 

Triterpenoids are also very common and diverse in the family. In the genus *Euphorbia*, different types of skeletons have been described, for example, tirucallane, euphane, lanostane, cycloartanes, lupane, oleanane, taraxarane, friedoursane, friedelane, and ursane [52]. And still, nowadays, new compounds are being discovered. A new cycloartane-type triterpene (23 R/S-3b-hydroxycycloart-24-ene-23-methyl ether) was isolated from the aerial parts of *Euphorbia dendroides* L [53]. In the genus *Croton*, several triterpenoids have been described [46]. For example, lupeol has been isolated from different parts of the species *Croton sylvaticus* Hochst. and *Croton zambezicus* Müll. Arg. [54,55]. β-Sitosterol has been isolated from *Croton zambezicus* Müll. Arg. and *Croton steenkampianus* Gerstner [15,56]. Betulinic acid, betulin, and lupenone have been isolated from the fruits of *Croton zambezicus* [57]. Jatrogrossidione, a triterpenoid isolated from the branches and leaves of *Jatropha gossypiifolia* L., was isolated by Zhan et al. [58]. 

Also, a few sesquiterpenoids have been reported from *Euphorbia*, *Croton*, *Jatropha*, etc. [46,59,60]. Aryanin, a new sesquiterpene lactone, was isolated from the aerial parts of *Euphorbia microsphaera* Boiss by Azizi et al. [61]. Another sesquiterpene, caryophyllene, was reported to be present in *Croton* species as a volatile constituent [46]. 

The presence of flavonoids in this family is widespread. These compounds could have an impact on health, for example, as potential anticancer agents that have been previously reported [62]. For example, quercitrin has been isolated from *Euphorbia hirta* L. [63], and apigenin has been isolated from *Croton betulaster* Mull, *Jatropha gossypiifolia* L., and *Macaranga gigantifolia* Merr. [17,20,64]. 

Some alkaloids have been isolated from this family also, from latex, roots, or other parts of the plants. Alkaloids are important chemicals; some of them have been isolated or developed for cancer treatment, such as the alkaloids of vinca [65]. For example, Novello et al. [66] have isolated, from *Croton echioides* Baill, a new alkaloid (*N-trans*-feruloyl-3,5-dihydroxyindolin-2-one) as a mixture with other already known alkaloids (*N-trans-p*-coumaroyl-tryptamine, *N-trans-p*-coumaroyl-5-hydroxytryptamine, *N-trans*-4-methoxy-cinnamoyl-5-hydroxytryptamine, and *N-trans*-feruloyl-5-hydroxytryptamine. 

### 3.2. Cytotoxic and Anticancer Effects of Euphorbiaceae Extracts In Vitro Studies

Testing plant extracts is one of the first steps in the search for new compounds with potential biological properties, including an anticancer effect. Many articles confirm the preliminary cytotoxic properties of extracts from many medicinal plant families, which can induce apoptosis in many cancer lines. The Euphorbiaceae family is a source of many interesting compounds with broad medicinal applications. The first step of our analysis was to analyze the effect of plant extracts on cytotoxic effects in in vitro studies. Mesas et al. [67] showed that an ethanolic extract of *Euphorbia lathyris* seeds rich in polyphenols such as esculetin, euphorbetin, gaultherin, and kaempferol-3-rutinoside had an antiproliferative effect against colon cancer cell lines (T84 and HCT-15) and glioblastoma multiforme. The authors demonstrated that the induction of apoptosis is associated with overexpression of caspase-9 (casp-9), caspase-3 (casp-3), and caspase-8 (casp-8) and activation of autophagy. In another study, Sultana et al. [68] showed that an aqueous leaf extract of *Excoecaria agallocha* (L.), where the head compound was bergenin, effectively reduced the proliferation of SiHa cervical cancer cells by inducing autophagy and apoptosis in a coordinated manner, with simultaneous stimulation of mitophagy and cell cycle arrest in the G2/M phase. In contrast, Kwan et al. [69] showed that a methanolic extract of *Euphorbia hirta* exhibited significant inhibition of MCF-7 breast cancer cell survival through induction of apoptosis via a casp-3-independent pathway, activation of caspase-2, caspase-6, casp-8, and casp-9, accumulation of cells in the S and G2/M phases, and DNA fragmentation. Similarly, Mfotie Njoya et al. [70] showed that *Croton gratissimus* leaf extract exhibited cytotoxic effects on various cancer lines (A549, Caco-2, HeLa, MCF-7) and inhibited cancer cell growth through induction of caspase-3 (casp-3)/caspase-7 (casp-7) activation, with the highest induction (1.83-fold change) obtained on HeLa cells. Vargas-Madriz et al. [71] presented that *Cnidoscolus aconitifolius* and *Porophyllum ruderale*, rich in polyphenols, reduced the metabolic activity of human SW480 colon adenocarcinoma cells. In addition, both extracts increased the total number of apoptotic cells and arrested the cell cycle in the G0/G1 phases. Other studies are presented in Table 1.

### 3.3. Cytotoxic and Anticancer Effects of Euphorbiaceae Pure Compounds In Vitro Studies

Many studies have focused on investigating the induction of apoptosis through various signaling pathways in cancer cells as a result of isolated plant compounds. In their study, Wisniewski et al. [145] showed that, of the compounds derived from Euphorbia sp., latilagascene B is an effective *P*-glycoprotein inhibitor capable of increasing doxorubicin accumulation in resistant cells (human colon carcinoma LoVo cells). In contrast, Li et al. [146] showed that Trigothysoid N, a natural diterpenoid isolated from *Trigonostemon thyrsoideus*, revealed a strong ability to inhibit the proliferation of A549 lung cancer cells through cell cycle arrest. In addition, the compound can inhibit tumor proliferation and migration by targeting mitochondria, regulating the signal transducer and activator of transcription 3/focal adhesion quinase) signaling pathway (STAT3/FAK), and inhibiting angiogenesis. In another study, Lin et al. [147] showed that Euphorbia L2, a lathyrane diterpenoid isolated from the seeds of *Euphorbia lathyris* L., possessed potent cytotoxicity against A549 lung cancer cells and induced apoptosis through the mitochondrial pathway via an increase in reactive oxygen species (ROS), loss of mitochondrial potential, release of cytochrome c, activation of casp-9 and casp-3, and cleavage of poly(ADP-ribose) polymerase. In turn, Fan et al. [148] showed that the 8,9-seco-*ent*-kaurane diterpenoid isolated from *Croton kongensis* induced apoptosis, autophagy, and metastasis suppression in triple-negative breast cancer (TNBC) cells by inhibiting Akt. Additionally, in in vivo studies, it significantly inhibited TNBC tumor growth without causing side effects. Wongprayoon et al. [149] noted that *Euphorbia lactea* triterpenoid friedelan-3β-ol was cytotoxic to several cancer cell lines, including HN22, HepG2, HCT116, and HeLa. Furthermore, the authors showed that the compound induced S-phase cell cycle arrest in HN22 cells without inducing apoptosis at the same concentration and exposure time. Studies about isolated compounds are presented in Table 2 below. 

### 3.4. Anticancer Effects of Euphorbiaceae Extracts and Pure Compounds In Vivo Studies

The anticancer effects of both extracts and pure compounds have been tested in vivo using various animal models. Many studies are available on in vivo experiments on a number of plants from the Euphorbiaceae family. de Abrantes et al. [209] showed that Tonantzitlolone B (TNZ-B), a diterpene from *Stillingia loranthacea*, exhibits antitumor activity (1.5 or 3 mg/kg i.p.) in a mouse model of Ehrlich ascites carcinoma. The LD50 was estimated to be approximately 25 mg/kg (i.p.). TNZ-B reduced Ehrlich tumor volume and the total number of viable tumor cells. In addition, TNZ-B reduced the density of peri-tumor microvessels, suggesting an anti-angiogenic effect. Gowrav Adiga et al. [210] investigated the anticancer activity of a methanol extract of the stem bark of *Croton oblongifolius* in Sprague-Dawley rats. The rats were tumor-induced with dimethylbenz(a)anthracene (DMBA), administered orally and intramammarily, and with a plant extract. After obtaining the appropriate tumor mass, the extract was administered to the rats by gavage at 200, 500, and 800 mg/kg, which showed a dose-dependent reduction in mammary tumor volume, as confirmed by histopathological observations in the treated groups. In other studies, using a mouse model of breast cancer induced by 4T1 cells, Majumder et al. [211] demonstrated the effect of *Ricinus communis* fruit extract, rich in ricinine, *p*-coumaric acid, epigallocatechin, and ricinoleic acid, on breast cancer progression in vivo. Tumors were induced in female Balb/c mice by injecting 4T1 cells subcutaneously into the mammary fat pad. After 10 days, one group of animals received 4 i.p. doses of the extract (5 mg/kg body weight), while the other group received only the vehicle (0.9% saline). The tumor in the control group continued to grow, and animals treated with the extract showed a significant reduction in tumor volume over time. Tumors removed from the animals after 22 days showed a reduction in tumor volume of over 88% compared to the control group. Table 3 shows in vivo studies.

### 3.5. Anticancer and Cytotoxic Effects of Nanoparticles Prepared from Euphobiaceae Extracts and Pure Compounds

Nanoparticles (NP) combined with extracts or pure compounds of plant origin are currently a well-known method for biological research and enhancing the effects of various substances in in vitro or in vivo models. In the research by Ghramh et al. [218], an ethanolic leaf extract of *Ricinus communis* with gold nanoparticles (AuNPs) demonstrated a cytotoxic effect. Studies have shown that the extract in combination with AuNPs has a stronger effect on HeLa and HepG2 lines than the *Ricinus communis* extract alone. In turn, Alqahtani et al. [219] also demonstrated a stronger cytotoxic effect of *Jatropha pelargoniifolia* extract in combination with chitosan nanoparticles against human lung adenocarcinoma (A549) than the extract alone. The same author revealed that Euphorbia retusa with a combination of zinc oxide NPs (ZnONPs) exhibited cytotoxic activity against A549 (IC_50_ = 22.3 µg/mL), HepG2 (IC_50_ = 25.6), Huh-7 (IC_50_ = 25.7), MCF-7 (IC_50_ = 37.7), and MDA-MB-231 (IC_50_ = 37) [220]. Similarly, Aboulthana et al. [221] showed that the cytotoxic activity of *Croton tiglium* seed extract increased after the incorporation of ZnONPs against human colon cancer cells (CACO-2). Additionally, the extract combined with ZnONPs stopped the increase of CACO-2 in G2/M and increased the percentage of total apoptotic cells and necrosis. A novel approach using *Excoecaria agallocha* leaf extract for the synthesis of silver NPs (AgONPs) was demonstrated by Banerjee et al. [222] AgONPs exerted initial cytotoxicity specifically against all experimental malignant cells (murine melanoma (B16F10), murine colon cancer (CT26), murine lung adenocarcinoma (3LL), and murine Ehrlich ascites carcinoma (EAC)), while sparing normal cell lines. Furthermore, both in vitro and ex vivo, AgONPs are equally effective in inducing apoptosis in all cancer cells. Studies on nanoparticles are presented in Table 4 below.

## 4. Potential Anticancer Mechanism of Action of Euphorbiaceae Extracts and Isolated Compounds

**Figure 2:**
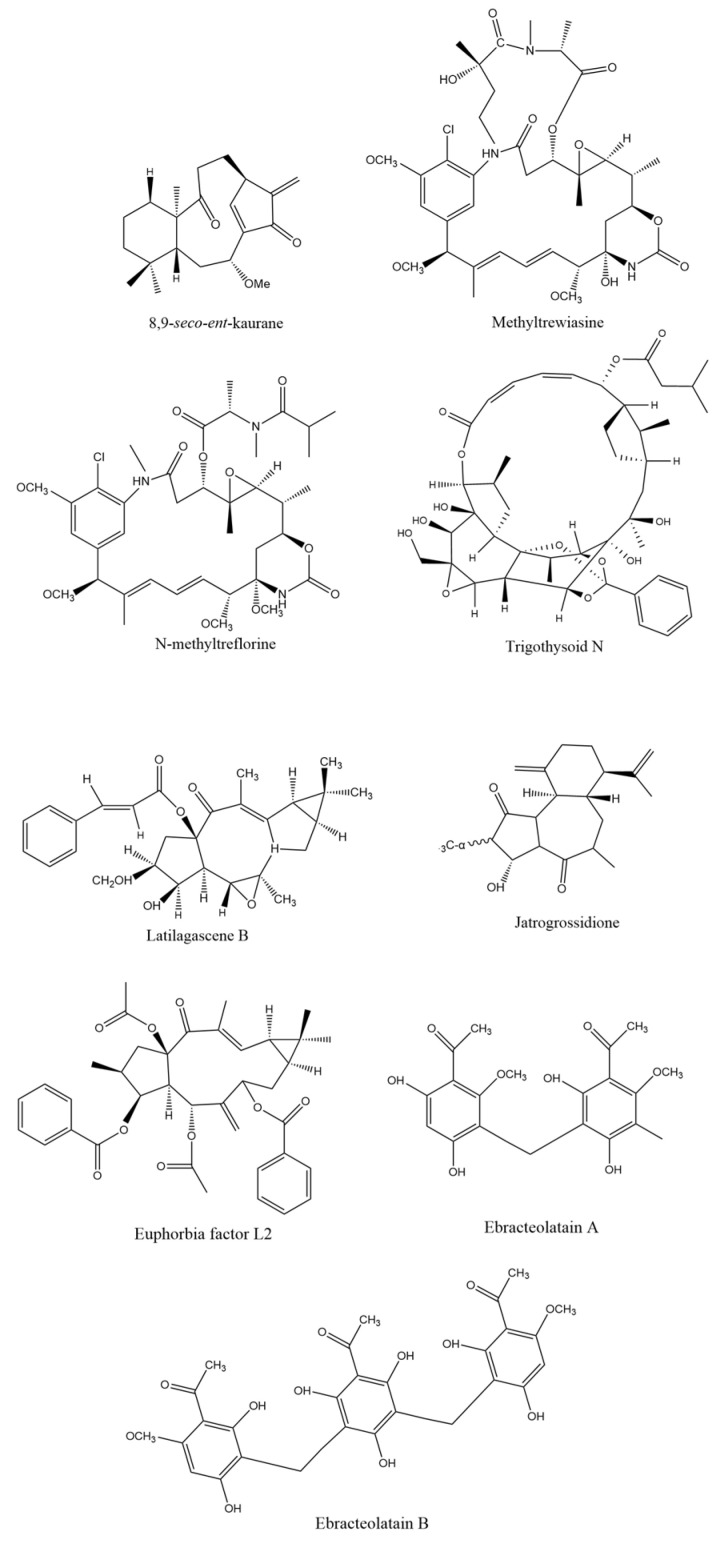
Chemical structure of isolated compounds from Euphorbiaceae plants with anticancer potential.

The cytotoxic activity reported by several authors in cancer cell lines treated with Euphorbiaceae extracts or isolated compounds may be due to different mechanisms of action. It is known that these extracts and compounds can activate the intrinsic pathway and the extrinsic pathway of apoptosis. The chemical structures of the main isolated compounds with anticancer activity and their underlying mechanisms of action are presented in Figure 2 below.

To activate the intrinsic pathway, the extract or compound could act as a ROS inductor. For example, *Croton gratissimus* Burch, *Drypetes sepiaria* (Wight & Arn.) Pax & K. Hoffm., *Euphorbia cactus* Ehrenb. ex Boiss., *Euphorbia hirta* L., *Euphorbia ingens* E. Mey. ex Boiss., *Euphorbia lathyrism* L., *Excoecaria agallocha* L., *Euphorbia helioscopia* L., and *Ricinus communis* L. extracts [67,68,70,94,95,115,211,215], and the pure compounds Euphorbia Factor L2, Ebracteolatain A, and Ebracteolatain B [147,232], activate the intrinsic pathway at some point. DNA damage promotes the liberation of cytochrome C by mitochondria. Then, cytochrome c and the APAF-1 (Apoptotic Protease Activating Factor-1) protein join together to activate casp-9. In this stage, it becomes a cascade release of different caspases activating each other from an inactive form to an active form and, at the end, activating casp-3, leading, finally, to apoptosis [67,70,94,136]. During this process, mitochondria also release SMAC/DIABLO (Second Mitochondria-Derived Activator of Caspase), an inhibitor of the XIAP (X-linked inhibitor of apoptosis) protein [233,234]. The caspases also cleave PARP (Poly ADP-Ribose Polymerase); this process is considered a hallmark of apoptosis [233].

The extrinsic pathway could be activated too, increasing the expression of casp-2/ casp-8. For example, some authors have reported this activity with *Croton gratissimus* Burch, *Drypetes sepiaria* (Wight & Arn.) Pax & K.Hoffm, and *Euphorbia lathyrism* L *extracts* [67,70,94].

The expression of p53 could also be affected by this family of plants. Several authors have reported higher levels of p53 after treatment with *Euphorbia pulcherrima* Willd. ex Klotzsch, *Euphorbia heterophylla* L., *Euhporbia ingens* E.Mey. ex Boiss, and *Excoecaria agallocha* L. in cancer cell lines [68,105,115]. p53 is a tumor-suppressive protein with the ability to induce cell death, including through apoptotic mechanisms and other transcription-dependent mechanisms or independent mechanisms [235]. Zhou et al. [232] also found that the activity of the pure compounds Ebracteolatain A and Ebracteolatain B increased p53 levels and decreased survivin levels. Survivin is a survival protein that inhibits caspases and blocks cell death [236]. Sultana et al. [68] found an altered expression on the levels of p21, the main target protein of p53. This protein, also known as CDKN1A (cyclin-dependent kinase inhibitor 1A), regulates the progression of the cell cycle with cyclin B. The balance of Bax/Bcl family protein expression can also be regulated through p53.

Changing the Bcl2/Bax (antiapoptotic/proapoptotic) protein balance could also be regulated by Euphorbiaceae plants through the STAT3 gene transcription pathway [146]. This balance is reported to be higher for Bax proteins than Bcl2 proteins when cancer cells are treated with *Croton tiglium* L., *Euphorbia cactus* Ehrenb. ex Boiss., *Euphorbia pulcherrima* Willd. ex Klotzsch, *Euphorbia heterophylla* L., *Euphorbia helioscopia* L., *Euphorbia hierosolymitana* Boiss., and *Ricinus communis* L. extracts [13,95,105,106,211,215]. Proapoptotic protein Bax is higher when cancer cells receive treatment with Trigothysoid N, Ebracteolatain A, and Ebracteolatain B also [146,232].

Li et al. [146] reported that cell invasion and migration could be regulated by Trigothysoid N through the FAK pathway. Once the compound inhibits FAK, the transcription of metalloproteinase (MMP)-2 and MMP-9 is downregulated, and the cells are not able to invade or migrate to other tissues.

**Figure 3:**
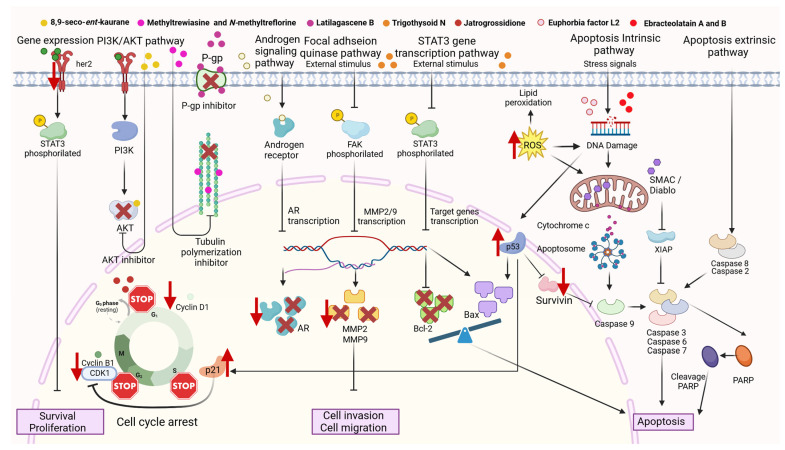
Potential anticancer mechanisms of action of Euphorbiaceae extracts and isolated compounds (created by BioRender).

The Euphorbiaceae extracts and isolated compounds could modulate the level of citoplasmatic proteins, acting as inhibitors or downregulating/upregulating them. For example, Okpako et al. [115] reported a downregulation in androgen receptors after the treatment of prostate cancer cells DU-145 with *Euphorbia ingens* E.Mey. ex Boiss extract. Lei et al. [206] reported inhibited tubulin polymerization after treatment with Methyltrewiasine and *N*-methyltreflorine. Tubulin polymerization/despolimerization plays a critical role in the cell cycle, and some important anticancer drugs are microtubule stabilizers of vegetal origin, for example, paclitaxel and docetaxel. Another example of protein regulation was reported by Shi et al. [237]. Using 8,9-seco-*ent*-kaurane diterpenoid isolated from *Croton kongensis* Gagnep in TNBC cells, the compound acted like an Akt inhibitor, which could induce apoptosis, autophagy, cell G2/M circle arrest, and inhibit cell migration. Latilagascene B was reported to be a *p*-glycoprotein inhibitor (Multidrug-resistant receptor) [145]. The cell cycle is regulated differently depending on the extract or pure compound used in cancer cells. In such a way that we can find stops in phase G1 [58,146], in phase G2/M [68,85], or in phase S [58] after the treatment with Euphorbiaceae. The anticancer potential mechanism of action of extracts and compounds from the Euphorbiaceae family is shown in Figure 3 below.

## 5. Conclusions and Future Perspectives

Research into the anticancer properties of plants in the Euphorbiaceae family shows promising potential. Cancer continues to be a global health problem, both in terms of health and economy. The lack of effectiveness and selectivity of the currently most used anticancer drugs makes it necessary to continue the search for new drugs. Nature continues to be an inexhaustible source of useful molecules to cure or alleviate diseases. It is for this reason that it is necessary to continue investigating the molecules present in living beings that surround us, including plants, algae, fungi, and marine organisms. In this review, we emphasize the usefulness of the various compounds present in the Euphorbiaceae family. This family of vascular plants is a very diverse family, both in genera and species and in secondary metabolites. The presence of different types of terpenoids, flavonoids, and alkaloids with cytotoxic activity against cancer makes us think about the number of possibilities that these compounds could offer alone or through the synthesis of different derivatives from them. All these active compounds represent an opportunity for the treatment of cancer; however, from a future perspective, it is necessary to continue researching all these molecules. Greater funding is necessary for the research of new molecules to be able to advance them more quickly from the process of discovery to the regulation and approval process for the market and patients. In this context, preclinical and clinical studies in animal models, followed by human clinical trials to evaluate their efficacy, safety, and dosage in different types of cancer, seem to play a very important role in this field.

## Figures and Tables

**Table 1:** Cytotoxic properties of selected extracts from the Euphorbiaceae family against cancer cells.

Name of the Species	Part of the Plant	Type of Extract	Class of Compounds/Compounds Identified in Extract	Cell Lines	IC_50_	Activity/ Mechanism/ Effect	Ref.
*Acalypha fruticosa* Forssk.	Aerial parts	Methanol	-	MCF-7, HCT-116, and HepG-2	12.2 ± 0.6 μg/mL, 4.81 ± 0.4 μg/mL, and 5.21 ± 0.7 μg/mL, respectively.	Cytotoxic	[72]
*Acalypha indica* L.	Leaves	Hexane	-	MCF-7	50 μg/mL	Cytotoxic	[73]
*Acalypha monostachya* Cav.	Aerial parts	Distilled water, absolute methanol, and *n*-hexane	Phenols, coumarins, lactones, flavonoids, saponins, aromatic compounds, carbohydrates, and carbonyl groups. Methanol and hexane: steroids and terpenoids. Aqueous: alkaloids.	HeLa and MDA-MB-231	-	Cytotoxic	[74]
*Baliospermum montanum* (Willd.) Müll.Arg.	Leaves	Methanol	-	Jurkat	298 μg/mL	Cytotoxic	[75]
*Baliospermum montanum* (Willd.) Müll.Arg.	Roots	Ethanol	Propiophenones	HepG2 and KKU M156	HepG2 (0.06 ± 0.02 μg/mL) and KKU M157 (0.16 ± 0.02 μg/mL)	Cytotoxic	[76]
*Blumeodendron toxbrai* (Blume.)	Stem Bark	Hexane, Dichloromethane, and Methanolic	-	MCF-7	Hexane extract 121.24 ± 0.15 µg/mL, Dichloromethane extract 55 ± 0.48 µg/mL, and methanolic extract 70.71 ± 0.15 µg/mL	Cytotoxic	[77]
*Croton sylvaticus* Hochst.	Leaves	Acetone and ethanol	-	A549, Caco-2, HeLa, and MCF-7	Acetone extract: A549 (32.78 ± 2.55 μg/mL), Caco-2 (150.63 ± 8.79 μg/mL), HeLa (169.09 ± 13.0 μg/mL), MCF-7 (13.13 ± 2.76 μg/mL). Ethanol extract: A549 (1.75 ± 0.62 μg/mL), Caco-2 (103.73 ± 1.47 μg/mL), HeLa (106.52 ± 4.50 μg/mL), MCF-7 (6.02 ± 1.60 μg/mL).	Cytotoxic. casp-3/casp7 pathway.	[70]
*Chrozophora oblongifolia* (Delile) Spreng.	Root Bark	Aqueous methanol, fractions with *n*-hexane, methylene chloride, and ethyl acetate	Carbohydrates and/or glycosides, sterols and/or triterpenes, tannins, alkaloids, and saponins	MCF-7 and Huh-7	Cytotoxic activity (%) of the methanolic extracts MCF-7 (15.82 ± 0.66) and Huh-7 (31.28 ± 0.68). *n*-hexane MCF-7 (59.55 ± 0.76) and Huh-7 (54.17 ± 0.46). Methylene chloride, MCF-7 (83.83 ± 0.37) and Huh-7 (82.79 ± 0.55). Ethyl acetate MCF-7 (22.25 ± 0.13) and Huh-7 (72.52 ± 0.23).	Cytotoxic	[78]
*Chrozophora plicata* (Vahl) A.Juss. ex Spreng.	Leaves	Petroleum ether, chloroform, hexane, ethyl acetate, methanol, and water	flavonoids, alkaloids, glycosides, and lignans.	DAL	25–50 μg/mL	Cytotoxic	[79]
*Cnidoscolus aconitifolius* (Mill.) I.M. Johnst.	Leaves	Methanol	Saponins, tannins, terpenes, and flavonoids	MCF-7 and NCI-H460	4.50 ± 0.58 μg/mL and 3.29 ± 4.57 μg/mL, respectively.	Cytotoxic	[80]
*Cnidoscolus chayamansa* McVaugh.	Leaves	Ethanolic	-	HT-29	CTC50 (μg/mL) ≥ 1000 ± 0.00	Cytotoxic	[81]
*Cnidoscolus multilobus* (Pax) I.M. Johnst	Leaves	Ethanol-water (70:30)		HeLa	130–160 µg/mL	Cytotoxic	[82]
*Cnidoscolus quercifolius* Pohl	Bark root	Methanol, chloroform fraction	Faveline, Faveline methyl ether, Deoxofaveline, and Neofavelanone	OVCAR-8, HCT-116, and HL-60	OVCAR-8 15.23 ± 2.0 μg/mL, HCT-116 7.07 ± 0.59 μg/mL, and HL-60 4.95 ± 0.19 μg/mL	Cytotoxic	[83]
*Croton acutifolius* Esser	Twigs and leaves	Hexane, ethyl acetate, and methanol	Retusin	KKU-M213, FaDu, HT-29, MDA-M, B-231, SH-SY5Y, A 549 and MMNK-2	Ethyl acetate extract against KKU-M213, MDA-MB-231, A-549, and MMNK-1 with the ED50 at 3.31 μg/mL, 0.58 μg/mL, 0.58 μg/mL, and 0.65 μg/mL, respectively.	Cytotoxic	[84]
*Croton bonplandianus* Baill.	Leaves	Acetone	-	A549	15.68 ± 0.006 μg/mL	Cytotoxic, apoptosis, and G2M phase arrest	[85]
*Croton caudatus* Geiseler	Leaves	Chloroform, ethanol and aqueous	-	HeLa	80 μg/mL	Cytotoxic, increased DNA damage	[86]
*Croton caudatus* Geiseler	Leaves	Chloroform, ethanol, and aqueous	Alkaloids, saponins, tannins, and cardiac glycosides.	Dalton’s lymphoma (DL)	28.36 μg/mL	Cytotoxic	[87]
*Croton caudatus* Geiseler	Leaves	Methanol		HeLa	59.70 μg/mL	Cytotoxic	[88]
*Croton fluviatilis* Esser	Stems	Hexane, ethyl acetate, and methanol	β-amyrin (1), stigmasterol, and β-sitosterol	KKU-M213, FaDu, HT-29, MDA-M, B-231, SH-SY5Y, A-549, and MMNK-1	Hexane extract cytotoxicity against KKU-M213, MDA-MB-231, and A-549 at the ED50 values of 1.70 μg/mL, 2.62 μg/mL, and 0.60 μg/mL, respectively	Cytotoxic	[84]
*Croton heliotropiifolius* Kunth	Leaves	Methanol	Gallic acid	NCI-H292, MCF-7, Hep-2, and HL-60	% inhibitor activity in NCI-H292 (46.5 ± 2.6), MCF-7 (21.7 ± 3.7), Hep-2 (26.7 ± 7.1), and HL-60 (59.5 ± 2.9)	Inhibitory activity	[89,90]
*Croton membranaceus* Mϋll. Arg.	Roots	Hydroethanolic	5-Hydroxypipecolic acid, Phenol, 3, 5-bis(1, 1-dimethylethyl)-, 2-Octenoic acid, 5, 5, 7-trihyroxy, 9, 10-Secocholeasta-5,7,10(19)-tiene-1,3-diol, 25-[(trimethylsilyl)oxy]-, Phenol, 4-(3-hydroxy-1-propenyl)-2-methoxy, *n*-Hexadecanoic acid, Benzene, 1,2,4,5-tetrakis(1-methylethyl)-, Prednisolone acetate, 1H, 4H-Pyrazolo[3,4-b]pyran-5-carbonitrile, 6-amino-4-(2, 4, 5-trimethoxyphenyl)-3-methyl and Astaxanthin	22Rv1	3.809 μg/mL	Cytotoxic, inhibit colony-forming and migration abilities	[91]
*Croton sphaerogynus* Baill.	Leaves	Hexane, dichloromethane, and methanol	Abieta-8,11-diene-3-one, Podocarp-7-ene,13-methyl-13-vinyl-3-one, Abieta-8,11,13-trien12-ol, Podocarp-7-ene-3-ol, 13-methyl-13-vinyl, 13-Hydroxy-abieta8,11-dien-7-one, and Crotonin derivative	786-0, HT-29, K562, NCI-ADR/RES, NCI-H460, MCF-7, PC-3, OVCAR-3, U251, and UACC-62.	Hexane and dichloromethane against NCI-H460 (GI50 0.26 µg/mL and 0.33 µg/mL, respectively) and K562 (GI50 0.60 µg/mL and <0.25 µg/mL, respectively).	Antiproliferative	[92]
*Croton thorelii* Gagnep.	Stems	Hexane, ethyl acetate, and methanol	5-hydroxy-7,4′-dimethoxyflavone	KKU-M213, FaDu, HT-29, MDA-M, B-231, SH-SY5Y, A-549, and MMNK-3	KKU-M213, MDA-MB-231, and A-549 had ED50 values of 0.55 μg/mL, 0.72 μg/mL, and 1.75 μg/mL, respectively.	Cytotoxic	[84]
*Croton tiglium* L.	Seeds	Ethylether and methanol	Isoguanosine, 12-O-Acetylphorbol-13-tigliate, and 13-O-Acetylphorbol-20-linoleate	A549	-	Apoptosis via apoptosis regulator or bcl-2-like protein 4/B-cell lymphoma 2 protein (Bax/Bcl-2) Pathways	[13]
*Croton urucurana* Baill	-	Hydroalcoholic	-	U937 and THP1	402.2 μg/mL and 360.3 μg/mL, respectively.	Cytotoxic and Apoptosis	[93]
*Drypetes sepiaria* (Wight & Arn.) Pax & K.Hoffm.	Leaves	Methanol	Phenolics and flavonoids	SiHa	10 μg/mL	Cytotoxic, apoptosis, or necrosis. casp-3 activation.	[94]
*Euphorbia cactus* Ehrenb. ex Boiss.		Methanol	Phenols, flavonoids, diterpenes, sesquiterpenoids, terpenoids, anthocyanins, tannins, steroids, cerebrosides, anthraquinones, phloracetophenones, glycerols, alkaloids, and carbohydrates	A549, LoVo, and MCF-7	A549 (20.1 ± 0.5 μg/mL), LoVo (53.2 ± 0.4 μg/mL), and MCF-7 (58.80 ± 1.83 μg/mL)	Cytotoxic, in A549, G2M cell cycle arrest. changes in the level of gene expression of Bax, Bcl-2, and casp-3	[95]
*Euphorbia caducifolia* Haines	Aerial parts	Ethanol and fractions: aqueous, ethyl acetate, and petroleum ether.	Friedooleanane-3- ol, (3α)-, 1,2-Benzendicarboxylic acid, mono (2- ethylhexyl) ester, Docosanoic acid, methyl ester; methyl behenate, Hexadecanoic acid methyl ester, methyl palmitate, 9, 12-Octadecadienoic Acid (Z,Z)-, methyl ester; methyl Linoleate (Z,Z)- isomer 9-Octadecenoic acid (z)-, methyl ester; methyl oleate	MCF-7, NCI-H460, PC-3, and HeLa	MCF-7 (61 ± 1.0 μg/mL), NCI-H460 (19 ± 6.3 μg/mL), PC-3 (135 ± 3 μg/mL), and HeLa (80 ± 2 μg/mL)	Cytotoxic	[96]
*Euphorbia davidii* Subils	Whole plant	*n*-Hexane and chloroform	kaempferol 3-O-rhamnoside, myricetin 3-O-rhamnoside, and quercetin 3-O-rhamnoside.	HeLa, MCF7, A2781, and A431	GI %: *n*-Hexane extract against HeLa (22.44 ± 2.66), MCF7 (45.88 ± 1.58), A2780 (26.72 ± 0.91) and A431 (25.65 ± 2.99) and chloroform extract against HeLa (22.28 ± 2.74), MCF7 (52.63 ± 0.88), A2781 (47.10 ± 0.68) and A431 (21.64 ± 0.37)	Cytotoxic	[97]
*Euphorbia dendroides* L	Aerial parts	Ethanol	Ellagic acid, pyrogallol, e-vanillic acid, benzoic acid, catechin, epi-catechin, alpha-coumaric acid, and salicylic acid	HepG2, HCT116 and MCF	HepG2 (9.5 mg/mL), HCT-116 (13.6 mg/mL), and MCF-7 (20.9 mg/mL).	Antiproliferative	[98]
*Euphorbia graminea* Jacq.	Leaves	Aqueous Methanol, fractions with *n*-hexane, ethyl acetate, chloroform, and water	Tannins, flavonoids, saponins, cardiac glycosides, and terpenes	MCF7 and NCI-H460	At 250 µg/mL, the extract recorded −3.1 and +75% cytotoxic and growth inhibitory effects on MCF-7 and NCI-H460 cell lines, respectively. While the cytotoxic effect became more pronounced on MCF-7 cell lines as −2.19 and −9.6% cytotoxicities were recorded by the chloroform fraction at 75 and 100 µg/mL, the ethyl acetate fraction recorded +58.72 and +89.33% inhibitory effects at similar concentrations.	Cytotoxic	[99]
*Euphorbia grandicornis* Blanc	Aerial parts	Dichloromethane	Methyl 2,5-dihydroxybenzoate, *n*-octylbenzoate, Friedelanol, Germanicol, β-glutinol, β-amyrin, Stigmasterol, β-Sitosterol, (24R)-tirucalla-8,25-diene-3β, 24-diol, Euphorbol, Hexyl (E)-3-(4-hydroxy-3-methoxyphenyl)-2 propenoate	HeLa	83.84 ± 2.94 μg/mL	-	[100]
*Euphorbia grandicornis* Blanc	Roots and aerial parts	Dichloromethane	β-glutinol, β-amyrin, 24-methylenetirucalla-8-en-3β-ol, (−)-tirucalla-8, 25-diene-3β-24R-diol, Stigmasterol, Sitosterol, Hexyl (E)-3-(4-hydroxy-3-methoxyphenyl)-2-propenoate	MCF-7 and HCC70	For roots, extract MCF-7 (0.83 ± 1.14 μg/mL) and HCC70 (0.83 ± 1.14 μg/mL), and for aerial parts, extract MCF-7 (1.03 ± 1.15 μg/mL), and HCC70 (0.31 ± 1.06 μg/mL)	Cytotoxic	[101]
*Euphorbia grantii* Oliv.	Aerial parts	Methanol and dichloromethane fraction	Friedelin, 3-b-firedelinol, epifriedelanol, euphol, cycloartenol, cycloartenyl acetate, epifriedelinyl acetate, and euphylbenzoate	MCF-7 and MCF-7ADR	Methanol extracts MCF-7 (16.47 μg/mL) and MCF-7ADR (19.55 μg/mL)Dichloromethane fraction, MCF-7 (10.31 μg/mL) and MCF-7ADR (10.41 μg/mL)	Cytotoxic	[102]
*Euphorbia granulata* Forssk.	Whole plant	Methanol	t-cinnamic acid, *p*-hydroxybenzoic acid, vanillic acid, 3,4-dihydroxybenzoic acid, syringic acid, *p*-coumaric acid, gallic acid, ferulic acid, caffeic acid, and sinapic acid	MCF-7, A2780 and HT-29	MCF-7 (16.23 ± 4.50 μg/mL), A2780 (22.80 ± 1.55 μg/mL), and HT-29 (41.89 ± 0.07 μg/mL)	Cytotoxic	[103]
*Euphorbia helioscopia* L.	Powdered	Hexane, acetone, methanol, and water		DLD-1	140.83 ± 0.31 μg/mL	Cytotoxic	[104],
*Euphorbia helioscopia* L.	Whole plant	Ethanol	-	Hep-2, T-47D, HT-29, and PC-3	%GI Hep-2 (27), T-47D (7), HT-29 (0), and PC-3 (11)	Cytotoxic	[12]
*Euphorbia heterophylla* Desf.	-	Methanol		HepG2	-	cell cycle arrest	[105]
*Euphorbia hierosolymitana* Boiss.	Whole plant	Methanol		MCF-7, HepG-2, HCT-116, PC-3	HCT-116 (4.22 ppm)	Cytotoxic. Changed the cell cycle and affected the gene expression of her2, Bax, and Bcl-2.	[106]
*Euphorbia hierosolymitana* Boiss.	Aerial parts	Ethyl acetate and *n*-butanol fraction	2-Myristynoyl acid pantethene, Palmitic acid, methyl ester, Pyrrolidine,1-bicyclo 3,2,1Oct-2-En-3-Yl, Desulphosinigrin	MCF-7, PC3, A549 and Caco-2.	MCF-7 (93 μg/mL), PC3 (100 μg/mL), A549 (103 μg/mL), and Caco-2 (170 μg/mL). For the *n*-butanol fraction, for MCF-7, PC3, and A549 (>500 μg/mL), and for Caco-2 (152 μg/mL).	Cytostatic	[107]
*Euphorbia hirta* L.	Leaves	Ethanol	Anthroquinone, terpenoids, alkaloids, phenolic compounds, tannins, flavonoids, steroids, coumarins, and saponins	DLA and EAC	DLA (560.83 µg/mL) and EAC (384.7 μg/mL)	Cytotoxic	[108]
*Euphorbia hirta* L.	Roots	Ethanolic	-	MCF-7	From 10 µg/mL (61.57 ± 0.16 µg/mL) to 100 µg/mL (48.08 ± 0.30 µg/mL)	Cytotoxic	[109]
*Euphorbia hirta* L.	Whole plant	Ethanol	9,12,15-Octadecatrien-1-ol, Pentadecylic acid, Ethyl palmitate, Methyl linoleate, 5-Hydroxymethyl-2-furancarboxaldehyde, Ethyl linoleolate	HL-60	50–100 μg/mL	Anticancer	[110]
*Euphorbia hirta* L.	Whole plant	Petroleum Ether and Chloroform	-	HepG2	Petroleum ether 200 μg/mL and Chloroform 150 μg/mL	Cytotoxic	[111]
*Euphorbia hirta* L.	Whole plant	Methanol and distilled water	-	HCT-15	510.66 μg/mL	Cytotoxic	[112]
*Euphorbia hyssopifolia* L.	Aerial parts	Ethanol	Mono and sesquiterpenes, triterpenes, steroids, flavonoids, cynnamic derivatives, hydrolysable tannins, and saponnins	HepG2	-	Cytotoxic and genotoxic	[113]
*Euphorbia inarticulata* Schweinf.	-	Ethanolic	Catechol, syringic acid, cinnamic acid, caffeic acid, gallic acid, ellagic acid, and benzoic acid	Huh-7 and HeLa	Huh-7 104.52 ± 2.74 μg/mL and HeLa 145.11 ± 6.21 μg/mL	Cytotoxic	[114]
*Euphorbia ingens* E.Mey. ex Boiss.	Root	Dichloromethane:methanol (36.25%) and ethyl acetate fraction	Mayor constituents: 6-pentylidene-4,5-secoandrostane-4,17 beta-diol, 2-bornanol, 1-octadecene, 1-tridecene, and 1-dodecene	DU-145	9.71 ± 0.40 μg/mL	Cytotoxic, Regulation of the Phosphoinositide 3-kinases/Protein kinase B (PI3K/Akt), MAPK, and tumor protein p53 (p53) signalling pathways	[115]
*Euphorbia lactea* Haw.	Stems	Hydroalcoholic	-	HN22	250–500 μg/mL	Cytotoxic and anti-migratory activity	[116]
*Euphorbia macroclada* Boiss.	Leaves, flower and body	Acetone	-	MCF–7 and L-929	Leaves (8.91 ± 0.10 μg/mL)	Cytotoxic	[117]
*Euphorbia milii* Des Moul.	Aerial parts	Methanol	-	HepG-2	HepG-2 (87.1 ± 9.4 μg/mL)	Cytotoxic	[118]
*Euphorbia nivulia* Buch.-Ham	Aerial parts	Aqueous ethanol	Phenols, flavonoids, terpenoids, glycosides, alkaloids, saponins, steroids, and tannins	HeLa cells	-	Cytotoxic	[119]
*Euphorbia paralias* L.	Aerial parts	Methanol, fractions with dichloromethane, and ethyl acetate	Gallic acid, ellagic acid, Kaempferol-3-O-(600-O-galloyl-b-D-glucopyranoside), Quercetin-3-O-b-D-glucopyranoside, and Quercetin-3-O-b-D-arabinopyranoside	HepG2	HepG2 (26.4 ± 1.2 mg/mL)	Cytotoxic	[120]
*Euphorbia platyphyllos* L.	Whole plant	Diethyl ether, petroleum ether, ethyl acetate, methanol, water infusion, and water decoction	-	MCF-7	>300 μg/mL, 98.07 ± 0.58 μg/mL, 46.24 ± 0.57 μg/mL, 97.16 ± 0.51 μg/mL, 38.29 ± 0.57 μg/mL and 27.79 ± 0.58 μg/mL respectively.	Cytotoxic, Apoptosis	[121]
*Euphorbia pulcherrima* Willd. ex Klotzsch	-	Methanol		HepG2	-	cell cycle arrest	[105]
*Euphorbia rigida* M.Bieb.	Aerial parts	Methanol	-	Hep3B and HepG2	-	Cytotoxic	[122]
*Euphorbia royleana* Boiss.	Aerial parts	Methanol	-	HepG-2, HCT-116 and MCF-7	HepG-2 (0.42 ± 0.7), and HCT-116 (285.1 ± 19.2)	Cytotoxic	[118]
*Euphorbia tirucalli* L.	Aerial parts	Ethanol and fractions with ethanol, hexane, dichloromethane, ethyl acetate, and aqueous fractions AGS	Aqueous and ethyl acetate fractions (ellagic acid, 1-o-Galloyl-β-d-glucose, sucrose or isomers, Quercitrin, 2,3-hexahydroxydiphenoyl-d-glucose or isomers, rutin, corilagin or isomers, and pedunculagin/casuariin)	AGS	AGS fractions: ethanol (11.73 ± 0.31 μg/mL), hexane (10.33 ± 2.01 μg/mL), dichloromethane (85.00 ± 9.5 μg/mL), ethyl acetate (120.9 ± 2.21 μg/mL), and aqueous (13.08 ± 0.99 μg/mL)	Cytotoxic	[123]
*Euphorbia tirucalli* L.	Aerial parts	Hexanic and hydroalcoholic	-	HCT-116	25.26 ± 0.18 μg/mL	Cytotoxic, Increase in the expression of casp-3 and p53	[124]
*Euphorbia triaculeata* Forssk.	Whole plant	Methanol	-	MCF-7, PC-3, HEPG2 and MCF-10A	0–50 μg/mL	Cytotoxicity and genotoxicity	[125]
*Euphorbia turcomanica* Boiss.	Aerial parts	Heptane, ethyl acetate, dichloromethane, acetone, methanol, and methanol-water (70–30)	Flavonoid, alkaloid, anthraquinone, and tannin.	Hela and HT-29	Methanol-water (50 μg/mL), acetone (90 μg/mL), dichloromethane (230 µg/mL), methanol (420 µg/mL), and heptane (450 µg/mL) in HeLa cells. Methanol-water (43 µg/mL), acetone (115 µg/mL), dichloromethane (125 µg/mL), methanol (250 µg/mL), and heptane (390 µg/mL) in HT-29 cells.	Cytotoxic	[126]
*Euphorbia umbellata* (Pax) Bruyns	Fresh stems and leaves	Methanol	-	U-251, MCF-7, 786-0, NCI-H460	Stems extract (GI50 between 8.1 and 30.3 mg/mL) Leaf extract (GI50 between 35.6 and >250 mg/mL). Acetone fraction (GI50, between 0.37 and 2.9 mg/mL)	Antiproliferative	[127]
*Euphorbia umbellata* (Pax) Bruyns	Bark	Ethanol:water (70:30), chloroform fraction	Euphol, sitosterol, lanosterol, lupeol, cycloartenol, friedelin-3b-ol, friedelin	Jurkat clone E6-1	29.00 ± 1.49 µg/mL, 10.06 ± 1.48 µg/mL and 4.83 ± 2.25 µg/mL for 24, 48 and 72 h	Cytotoxic, apoptosis and cell cycle arrest	[128]
*Euphorbia umbellata* (Pax) Bruyns	Green branches and leaves	Water, methanol, ethanol, and chloroform	Flavonoids, phenols and tannins, terpenoids, steroids, carbohdraates, alkaloids and saponins.	A549	Water (90.15 ± 2.50 µg/mL), ethanol (125.27 ± 2.00 µg/mL), chloroform (197.66 ± 2.40 µg/mL), and methanol (4.30 ± 0.44 µg/mL)	Cytotoxic	[129]
*Excoecaria agallocha* L.	Leaves	Methanol and Chloroform	-	Hep 2	Methanol extract range between 125–200 µg/mL and chloroform extract, 15–30 µg/mL	Cytotoxic	[130]
*Excoecaria agallocha* L.	Leaves	Methanol	-	M14, SKMEL5, SKMEL2, SKMEL28, MALME3M, UACC62, UACC257, U251, SNB19, MDA-MB-231, MCF-7, T47D, BT549, Ovcar3, Ovcar5, HCC2998, Colo205, HCT15, KM12, HeLa, SIHA and C33A	M14 (47.5 ± 4.12 μg/mL), SKMEL5 (89.2 ± 4.62 μg/mL), SKMEL2 (30.0 ± 2.12 μg/mL), SKMEL28 (29.8 ± 2.46 μg/mL), MALME3M (35.0 ± 3.12 μg/mL), UACC62 (34.0 ± 2.90 μg/mL), UACC257 (26.0 ± 3.12 μg/mL), U251 (19.44 ± 1.14 μg/mL), SNB19 (19.84 ± 1.60 μg/mL), MDA-MB-231 (15.56 ± 1.14 μg/mL), MCF-7 (20.24 ± 1.16 μg/mL), T47D (19.20 ± 0.15 μg/mL), BT549 (63.42 ± 5.12 μg/mL), Ovcar3 (39.44 ± 1.14 μg/mL), Ovcar5 (19.84 ± 0.60 μg/mL), HCC2998 (20.28 ± 0.56 μg/mL), Colo205 (12.0 ± 2.12 μg/mL), HCT15 (39.0 ± 3.82 μg/mL), KM12 (20.0 ± 1.70 μg/mL) HeLa (18.60 ± 2.20 μg/mL), SIHA (23.42 ± 1.90 μg/mL) and C33A (39.9 ± 3.10 μg/mL)	Cytotoxic	[131]
*Flueggea leucopyrus* Willd.	Leaves and bark	Ethyl acetate and methanol.	Bergenin and bergenin isomers	A2780	Methanol extract of bark 12.58 ± 1.02 µg/mL, Ethyl acetate extract of leaves 36.35 ± 0.17 µg/mL	Cytotoxic and antiproliferative effect	[132]
*Hura crepitans* L.	Leaves	Methanol and *n*-butanol	-	HepG2	-	Cytotoxic	[133]
*Jatropha curcas* L.	Leaves	Methanol	Hexadecanoic acid, hexadecanoic acid methyl ester, anethole, estragol, oleic acid, phytol, and carvacrol, octadecanoic acid methyl ester, and thymol	HepG2	47.2 ± 2.48 μg/mL	Cytotoxic	[11]
*Jatropha curcas* L.	Leaves	Ethanol	-	T-47D, SiHa, and OVCAR-7	%GI T-47D (0), SiHa (47), and OVCAR-7 (30)	Cytotoxic	[12]
*Jatropha dioica* Sesse ex Cerv.	Roots	Aqueous	Alkaloids, flavonoids, saponins, phenols, tannins, and carbohydrates		-		
*Jatropha gossypifolia* L.	Leaves	Methanol	-	HepG2	15.3 ± 0.95 μg/mL	Cytotoxic	[11]
*Jatropha multifida* L.	Leaves	Methanol	-	HepG2	29.6 ± 1.27 μg/mL	Cytotoxic	[11]
*Jatropha podagrica* Hook.	Leaves	Methanol and distilled water (80:20)	-	A549 and PC12	GI > 80% at 100 µg/mL	Antiproliferative	[11]
*Jatropha zeyheri* Sond.	Roots	Ethyl acetate	Mayor contents: Hexadecanoic acid, Octadecanoic acid, (Z)-9-Octadecenamide, 11-*n*-Decylheneicosane, Octacosane, 9-Hexacosene, Ethyl vallesiachotamate, Cyclooctacosane, Cyclotetracosane, and Tricosane	Caco-2, A547 and MCF-7	8.83 ± 0.00 μg/mL, 224.48 ± 0.01 μg/mL, and 102.88 ± 2.17 μg/mL respectively	Cytotoxic	[134]
*Mallotus cumingii* Müll.Arg.	Leaves	Methanol, fractions with hexane and ethyl acetate	Phenolic compounds, flavonoids, terpenoids, cardiac glicosides and saponins.	HCT-116	Methanol (31.51 μg/mL), hexane fraction (17.49 μg/mL) and ethyl acetate fraction (7.75 μg/mL)	Cytotoxic	[135]
*Mallotus phillippensis* (Lam.) Müll.Arg.	Leaves	Methanol	alkaloids, flavonoids, tannins, diterpenes, steroids, and phenolic compounds	MCF-7	190 g/mL	Cytotoxic and apoptosis through the intrinsic pathway	[136]
*Manihot esculenta* Crantz	Aerial parts	Ether and chloroform fraction	-	A-549	Inhibition ratio % (69.71 ± 1.18) at 50 μg/mL	Cytotoxic	[137]
*Mercurialis annua* L.	Aerial parts	Ethanolic	Kaempferol, Isorhamnetin, Quercetin, and Rutin	K562, MCF-7, Hela, and A562	%GI 100 µM K562 (28.52 ± 0.57), MCF-7 (20.74 ± 6.96), Hela (14.44 ± 2.16), and A562 (10.33 ± 2.75)	Antiproliferative	[138]
*Plukenetia volubilis* L.	Leaves	Methanol, ethanol, chloroform, hexane, and water	terpenoids, saponins, and flavonoids	HeLa, and A549	Inhibition of 40–50% rate.	Antiproliferative effect	[139]
*Ricinus communis* L	Leaves	Aqueous	-	A375	48 µg/mL.	Cytotoxic	[140]
*Ricinus communis* L	Stems and seeds	Ethanol	-	A549, HT-29, SW-20, SiHa, Hep-2, T-47D, OVCAR-5 and PC-3	Seed extract activity was 41%, 11%, 12%, and 14% against A549, OVCAR-5, PC-5 respectively. Stem extract activity was 9%, 31% and 40% activity against Hep-2, HT-29, and SiHa cell lines (100 µg/mL).	Cytotoxic	[141]
*Ricinus communis* L.	Stems	Ethanol	-	Hep-2, HT-29, SiHa, and OVCAR-6	%GI Hep-2 (9), HT-29 (31), SiHa (47), and OVCAR-6 (30).	Cytotoxic	[12]
*Ricinus communis* L.	Seeds	Ethanol	-	502713, A549, OVCAR-5, and PC-6	%GI 502713 (41), A549 (11), OVCAR-5(12) and PC-5(14).	Cytotoxic	[12]
*Ricinus communis* L.	Different part of the seeds (testa, tegmen, embryo, endosperm, etc)	Methanol	Phorbol esters	THP-1	testa extract in THP-1 (109.9 μg/mL)	Cytotoxic	[142]
*Schinziophyton rautanenii* (Schinz) Radcl.-Sm.	Root and bark	Aqueous and methanolic	Alkaloids, flavonoids, anthraquinones, coumarins and triterpenenes	TK10, MCF–7, and UACC-62	Aqueous (100–150 µg/mL) and methanolic (70–120 µg/mL) for both cell lines.	Cytotoxic	[143]
*Tragia involucrata* L.	Whole plant	Ethanol	Clionasterol, Squalene, 2-Ethylhexyl phthalate, Phytol, neophytadiene, ethyl palmitate, ethyl linolate, linolenic acid, viminalol, etc	YAC-1	-	Cytotoxic	[144]

Data not reported are represented by “-“.

**Table 2:** Cytotoxic properties of isolated compounds from the Euphorbiaceae family of plants against cancer cells.

Name of the Species	Part of the Plant	Type of Extract	Class of Compounds/ Compounds Identified in Extract	Cell Lines	IC50	Activity/ Mechanism/Effect	Ref.
*Croton crassifolius* Geiseler	Roots	Ethanol	Crotonpyrone A	HeLa and NCI-446	HeLa (10.21 μg/mL) and NCI-446 (6.59 μg/mL)	Cytotoxic	[150]
*Croton crassifolius* Geiseler	Roots	Ethanol	Crotonpyrone B	HeLa and NCI-446	HeLa (9.54 μg/mL) and NCI-446 (6.52 μg/mL)	Cytotoxic	[150]
*Croton damayeshu* Y.T.Chang	Twigs and leaves	Ethanol	Crodamoid H	A549	A549 (0.9 ± 0.6 μM)	Cytotoxic	[151]
*Croton damayeshu* Y.T.Chang	Twigs and leaves	Ethanol	Crodamoid I	A549 and HL-60	A549 (1.3 ± 0.2 μM) and HL-60 (2.4 ± 1.0 μM)	Cytotoxic	[151]
*Croton damayeshu* Y.T.Chang	Twigs and leaves	Ethanol	4α-deoxyphorbol-12-tiglate-13-isobutyrate	A549 and HL-60	A549 (1.9 ± 0.1 μM) and HL-60 (1.8 ± 0.8 μM)	Cytotoxic	[151]
*Croton echioides* Baill.	Stem bark	EtOH/H_2_O	*N-trans*-4-Methoxy-cinnamoyl-5-hydroxytryptamine	HCT-116	HCT-116 (86.8 μmol L^−1^)	Cytotoxic	[21]
*Croton floribundus* SPRENG.	Root bark	Hexane	*ent*-kaur-16-en-6a,19-diol	HCT-116, HL60, MDA-MB-435, and HCT-8	HCT-116 (12.1 μg/mL), MDA-MB-435 (14.3 μg/mL), and HCT-9 (13.5 μg/mL)	Cytotoxic	[152]
*Croton lachnocarpus* Benth.	Leaves	Ethanol	7b,15-dihydroxy-*ent*-abieta-8,11,13-trien-3-one	A549, BGC-823, HepG2, HL-60, MCF-7,	A549 (56.3 μM), BGC-823 (68.7 μM), HepG2 (66.9 μM), HL-60 (52.3 μM), MCF-7 (56.2 μM), W480 (59.4 μM)	Cytotoxic	[49]
*Croton lachnocarpus* Benth.	Leaves	Ethanol	2b,15-dihydroxy-*ent*-abieta-8,11,13-triene	A549, BGC-823, HepG2, HL-60, MCF-7, W480	A549 (52.3 μM), BGC-823 (52.2 μM), HepG2 (59.7 μM), HL-60 (49.4 μM), MCF-7 (60.1 μM), W480 (57.6 μM)	Cytotoxic	[49]
*Croton lachnocarpus* Benth.	Leaves	Ethanol	7b,13a,15-tri-hydroxy-*ent*-abieta-8(14)-en-3-one	A549, BGC-823, HepG2, HL-60, MCF-7, W480	A549 (28.7 μM), BGC-823 (26.6 μM), HepG2 (25.3 μM), HL-60 (31.7 μM), MCF-7 (27.1 μM), W480 (24.9 μM)	Cytotoxic	[49]
*Croton laui* Merr. & F.P.Metcalf			crotonolide A	HL-60 and P-388	HL-60 (9.42 μM) and P-388 (7.45 μM)	Cytotoxic	[153]
*Croton tiglium* L.	Seeds	Ethanol	4-deoxy-20-oxophorbol 12-tiglyl 13-acetate	K562, A549, and Huh-7	K562 (0.03 μM), A549 (6.88 μM), and Huh-7(3.85 μM)	Cytotoxic	[154]
*Croton tiglium* L.	Seeds	Ethanol	7-oxo-5-ene-phorbol-13-(2-methylbutyrate	K562, A549, and Huh-7	K562 (0.03 μM), A549 (6.33 μM), and Huh-7(20.9 μM)	Cytotoxic	[154]
*Croton tiglium* L.	Seeds	Ethanol	crotignoid C	K562, A549, and Huh-7	K562 (0.07 μM), A549 (8.86 μM), and Huh-7(11.6 μM)	Cytotoxic	[154]
*Croton tiglium* L.	Seeds	Ethanol	13-O-(2-metyl)butyryl-phorbol	K562, A549, and Huh-7	K562 (0.05 μM), A549 (43.5 μM), and Huh-7(34.2 μM)	Cytotoxic	[154]
*Croton tiglium* L.	Seeds	Ethanol	12-O-tiglylphorbol-13-acetate	K562, A549, and Huh-7	K562 (0.07 μM), A549 (8.50 μM), and Huh-7(3.36 μM)	Cytotoxic	[154]
*Croton tiglium* L.	Seeds	Ethanol	crotignoid F	K562, A549, and Huh-7	K562 (0.05 μM), A549 (3.11 μM), and Huh-7(4.41 μM)	Cytotoxic	[154]
*Croton tiglium* L.	Seeds	Ethanol	phorbol	K562, A549, and Huh-7	K562 (0.10 μM), A549 (15.3 μM), and Huh-7(5.93 μM)	Cytotoxic	[154]
*Croton tiglium* L.	Seeds	Methanol	13-O-acetylphorbol-20-oleate.	SNUB387	SNUB387 (1.2 ± 0.1 μM)	Cytotoxic	[155]
*Croton tiglium* L.	Seeds	Methanol	13-O-acetyl-4-deoxy-4α-phorbol-20-linoleate.	SNUB387	SNUB387 (8.1 ± 1.3 μM)	Cytotoxic	[155]
*Croton tiglium* L.	Seeds	Methanol	13-O-acetyl-4-deoxy-4α-phorbol-20-oleate.	SNUB387	SNUB387 (6.6 ± 0.9 μM)	Cytotoxic	[155]
*Croton tiglium* L.	Branches and leaves	Ethanol	12-O-acetylphorbol-13-isobutyrate	K562, MOLT-4, U937, MCF-7, Hela, DU145, A549, SGC-7091, H1975, HL60	K562 (4.0 μM), MOLT-4 (2.4 μM), U937 (6.8 μM), MCF-7 (13 μM), Hela (3.9 μM), DU145 (7.2 μM), A549 (5.8 μM) SGC-7091 (13 μM), H1975 (10 μM), HL60 (12 μM)	Cytotoxic	[156]
*Croton tiglium* L.	Branches and leaves	Ethanol	12-O-benzoylphorbol-13-(2-methyl)butyrate	K562, MOLT-4, U937, MCF-7, Hela, DU145, A549, SGC-7091, H1975, HL60	K562 (15 μM), MOLT-4 (12 μM), U937 (17 μM), MCF-7 (20 μM), Hela (4.6 μM), DU145 (4.3 μM), A549 (6.9 μM), SGC-7091 (10 μM), H1975 (3.3 μM), HL60 (6.8 μM)	Cytotoxic	[156]
*Croton tiglium* L.	Branches and leaves	Ethanol	12-O-tiglyl-7-oxo-5-ene-phorbol-13-(2-methylbutyrate)	K562, MOLT-4, U937, MCF-7, Hela, DU145, A549, SGC-7091, H1975, HL60	K562 (17 μM), MOLT-4 (4.8 μM), U937 (21 μM), MCF-7 (20 μM), Hela (5.0 μM), DU145 (10 μM), A549 (19 μM), SGC-7091 (23 μM), H1975 (10 μM), HL60 (10 μM)	Cytotoxic	[156]
*Croton tiglium* L.	Branches and leaves	Ethanol	13-O-(2-metyl)butyryl-4-deoxy-4a-phorbol.	K562, MOLT-4, U937, MCF-7, Hela, DU145, A549, SGC-7091, H1975, HL60	K562 (8 μM), MOLT-4 (9.9 μM), U937 (18 μM), MCF-7 (24 μM), Hela (10 μM), DU145 (10 μM), A549 (4.5 μM), SGC-7091 (5.4 μM), H1975 (3.3 μM), HL60 (9.8 μM)	Cytotoxic	[156]
*Croton tiglium* L.	Branches and leaves	Ethanol	12-O-tiglylporbol-13-propionate	K562, MOLT-4, U937, MCF-7, Hela, DU145, A549, SGC-7091, H1975, HL60	K562 (4.4 μM), MOLT-4 (1.1 μM), U937 (5.5 μM), MCF-7 (>50 μM), Hela (9.2 μM), DU145 (1.1 μM), A549 (32 μM), SGC-7091 (43 μM), H1975 (10 μM), HL60 (1.2 μM)	Cytotoxic	[156]
*Croton tiglium* L.	Branches and leaves	Ethanol	12-O-tiglylphor-bol-13-isobutyrate	K562, MOLT-4, U937, MCF-7, Hela, DU145, A549, SGC-7091, H1975, HL60	K562 (2.2 μM), MOLT-4 (1.0 μM), U937 (2.6 μM), Hela (10 μM), DU145 (5.0 μM), H1975 (10 μM), HL60 (1.2 μM)	Cytotoxic	[156]
*Croton tiglium* L.	Branches and leaves	Ethanol	12-O-tiglylphorbol-13-(2-methyl)butyrate	K562, MOLT-4, U937, MCF-7, Hela, DU145, A549, SGC-7091, H1975, HL60	K562 (7.2 μM), MOLT-4 (10 μM), U937 (14 μM), MCF-7 (>50 μM), Hela (10 μM), DU145 (11 μM), A549 (>50 μM), SGC-7091 (>50 μM), H1975 (10 μM), HL60 (9.9 μM)	Cytotoxic	[156]
*Croton tiglium* L.	Branches and leaves	Ethanol	tiglin A	K562, MOLT-4, U937, MCF-7, Hela, DU145, A549, SGC-7091, H1975, HL60	K562 (15 μM), MOLT-4 (12 μM), U937 (17 μM), MCF-7 (20 μM), Hela (4.6 μM), DU145 (4.3 μM), A549 (6.9 μM), SGC-7091 (10 μM), H1975 (3.3 μM), HL60 (6.8 μM)	Cytotoxic	[156]
*Croton tiglium* L.	Seeds	Acetone	7-Keto-12-O-tiglylphorbol-13-acetate	HL60 and A549	HL60 (6.22 ± 3.24 μg/mL) and A549 (18.0 ± 9.48 μg/mL)	Cytotoxic	[157]
*Croton tiglium* L.	Seeds	Acetone	Phorbol-12-isobutyrate	HL60 and A549	HL60 (0.22 ± 0.15 μg/mL) and A549 (0.74 ± 0.48 μg/mL)	Cytotoxic	[157]
*Croton tiglium* L.	Seeds	Acetone	12-O-Tiglylphorbol-13-acetate	HL60 and A549	HL60 (0.02 ± 0.01 μg/mL) and A549 (0.10 ± 0.03 μg/mL)	Cytotoxic	[157]
*Croton tiglium* L.	Seeds	Acetone	12-O-(2-methyl)-butyrylphorbol-13-aetate	HL60 and A549	HL60 (<0.01μg/mL) and A549 (0.01 ± 0.00 μg/mL)	Cytotoxic	[157]
*Croton tiglium* L.	Seeds	Acetone	12-O-tiglyl-phorbol-13-isobutyrate	HL60 and A549	HL60 (<0.01 μg/mL) and A549 (<0.01 μg/mL)	Cytotoxic	[157]
*Croton tiglium* L.	Seeds	Acetone	phorbol-12-tigliate	HL60 and A549	HL60 (83.1 ± 2.89 μg/mL) and A549 (38.6 ± 30.1 μg/mL)	Cytotoxic	[157]
*Croton tiglium* L.	Seeds	Acetone	phorbol-12-tetradecanoate	HL60 and A549	HL60 (3.14 ± 2.17 μg/mL) and A549 (4.71 ± 1.92 μg/mL)	Cytotoxic	[157]
*Croton tiglium* L.	Seeds	Acetone	phorbol-13-aetate	HL60 and A549	HL60 (80.9 ± 11.6 μg/mL)	Cytotoxic	[157]
*Croton tiglium* L.	Seeds	Acetone	phorbol-13-decanoate	HL60 and A549	HL60 (0.02 ± 0.02 μg/mL) and A549 (0.94 ± 0.01 μg/mL)	Cytotoxic	[157]
*Croton tiglium* L.	Seeds	Acetone	4-deoxy-4α-phorbol-13-acetate	HL60 and A549	HL60 (89.0 ± 0.76 μg/mL)	Cytotoxic	[157]
*Croton urucurana* Baill.	Bark	Ethanol	Orbitide [1−9-NαC]-crourorb A1	786-O, HT29, MCF7, ADR-RES, Hep-G2, and PC-03	786-O (18.69 ± 0.82 μg/mL), HT29 (37.28 ± 0.57 μg/mL), MCF7 (35.49 ± 2.59 μg/mL), ADR-RES (3.98 ± 0.20 μg/mL), Hep-G2 (41.31 ± 2.70 μg/mL) and PC-03 (29.80 ± 0.34 μg/mL)	Cytotoxic	[158]
*Croton velutinus* Baill.	Roots	Methanol	(E)-1-(7,8-epoxypropen) phenyl benzoate	B16F10, HL-60, HCT116, MCF-7, and HepG2	B16F10 (14.4 ± 0.5 μM), HL-60 (9.8 ± 2.6 μM), HCT116 (12.9 ± 1.8 μM), MCF-7 (6.8 ± 1.59 μM) and HepG2 (16.7 ± 0.7 μM)	Cytotoxic	[159]
*Croton velutinus* Baill.	Roots	Methanol	sellovicine B	B16F10, HL-60, HCT116, MCF-7, and HepG2	B16F10 (13.8 ± 01 μM), HL-60 (11.4 ± 3.6 μM), HCT116 (13.2 ± 1.0 μM), MCF-7 (11.1 ± 1.4 μM) and HepG2 (18.3 ± 1.8 μM)	Cytotoxic	[159]
*Drypetes hainanensis* Merr.	Leaves and stems	Ethanol	4β-hydroxy-23-nor-friedel-3-one	BEL-7402, A549, HL60	GI rates: BEL-7402(3.0%), A549 (9.7%), HL60 (4.1%)	Cytotoxic	[160]
*Drypetes hainanensis* Merr.	Leaves and stems	Ethanol	friedelin	BEL-7402, A549, HL60	HL60 (1.3%)	Cytotoxic	[160]
*Drypetes hainanensis* Merr.	Leaves and stems	Ethanol	friedelane-3,7-dione	BEL-7402, A549, HL60	BEL-7402 (4.6%), A549 (21.1%), HL60 (43.1%)	Cytotoxic	[160]
*Euphorbia ammak* Schweinf.	Leaves	Ethanol	euphol	HeLa	HeLa (9.25 mg/mL)	Cytotoxic	[161]
*Euphorbia ammak* Schweinf.	Leaves	Ethanol	α-glutinol	HeLa	HeLa (7.6 mg/mL)	Cytotoxic	[161]
*Euphorbia ammak* Schweinf.	Leaves	Ethanol	stigmasterol	HeLa	HeLa (10 mg/mL)	Cytotoxic	[161]
*Euphorbia balsamifera* Aiton	Aerial parts	Ethanol	Kampferol-3,40-dimethyl ether	HCT-116, HePG2, and MCF7	HCT116 (111.46 mM), HePG2 (42.67 mM) and MCF7 (44.90 mM)	Cytotoxic	[162]
*Euphorbia connata* Boiss.	Aerial flowering parts	Dichloromethane/Acetone	3,7,14,15-tetraacetyl-5-propanoyl-13(17)-epoxy-8,10(18)-myrsinadiene	MDA-MB-231 and MCF-7	MDA-MB-231 (24.53 ± 3.39 μM) and MCF-7 (37.73 ± 3.41 μM)	Cytotoxic	[163]
*Euphorbia connata* Boiss.	Aerial flowering parts	Dichloromethane/Acetone	3,7,10,14,15-pentaacetyl-5-butanoyl-13,17-epoxy-8-myrsinene	MDA-MB-231 and MCF-7	MDA-MB-231 (26.67 ± 1.41 μM) and MCF-7 (34.57 ± 2.12 μM)	Cytotoxic	[163]
*Euphorbia dendroides* L.	Aerial parts	Methanol	23 R/S-3b-hydroxycycloart-24-ene23-methyl ether	HepG2, Huh-7, KLM-1, 1321N1, HeLa	HepG2 (20.67 ± 0.72 μM), Huh-7 (16.24 ± 0.53 μM), KLM-1 (22.59 ± 0.94 μM), 1321N1 (25.99 ± 0.31 μM), HeLa (40.50 ± 3.14 μM)	Cytotoxic	[164]
*Euphorbia dendroides* L.	Aerial parts	Methanol	24-methylene cycloartan-3b-ol	HepG2, Huh-7, KLM-1, 1321N1, HeLa	HepG2 (10.93 ± 0.21 μM), Huh-7 (7.42 ± 0.16 μM), KLM-1 (21.48 ± 0.60 μM), 1321N1 (12.32 ± 0.58 μM), HeLa (13.68 ± 0.16 μM)	Cytotoxic	[164]
*Euphorbia dendroides* L.	Aerial parts	Methanol	cycloart-23-ene-3b,25-diol monoacetate	HepG2, Huh-7, KLM-1, 1321N1, HeLa	HepG2 (12.81 ± 0.73 μM), Huh-7 (<0.47 μM), KLM-1 (22.48 ± 0.64 μM), 1321N1 (25.17 ± 0.32 μM), HeLa (54.05 ± 1.11 μM)	Cytotoxic	[164]
*Euphorbia dendroides* L.	Aerial parts	Methanol	3b-hydroxy-cycloart-23-ene-25 methyl ether	HepG2, Huh-7, KLM-1, 1321N1, HeLa	HepG2 (12.72 ± 2.38 μM), Huh-7 (<0.44 μM), KLM-(<0.44 μM), 1321N1 (0.63 ± 0.15 μM), HeLa (3.7 ± 0.39 μM)	Cytotoxic	[164]
*Euphorbia dendroides* L.	Aerial parts	Methanol	24 R/S-3b-hydroxy-25-methylene Figure 1. Chemical structures of compounds 1–11, isolated from Euphorbia dendroides L. aerial parts. 830 A. R. HASSAN ET AL. cycloartan-24-ol	HepG2, Huh-7, KLM-1, 1321N1, HeLa	HepG2 (15.54 ± 1.95), Huh-7 (16.33 ± 1.22), KLM-1 (22.38 ± 1.29), 1321N1 (13.53 ± 0.33), HeLa (>4.52)	Cytotoxic	[164]
*Euphorbia denticulata* Lam	-	Acetone	taraxast-12-ene-3β,20,21(α)-triol	DU-145	DU-145 (12.2 ± 2.9 µM)	Cytotoxic	[165]
*Euphorbia denticulata* Lam	-	Acetone	cycloartane-3β,25-diol	DU-145	DU-145 (27.5 ± 4.9 µM)	Cytotoxic	[165]
*Euphorbia denticulata* Lam	-	Acetone	cycloartane-3β,24,25-triol	DU-145	DU-145 (18.3 ± 1.4 µM)	Cytotoxic	[165]
*Euphorbia ebracteolata* Hayata	Roots	Ethanol	Ebracteolata A	HL60, A549, SMMC-7721, MCF-7, and SW480	HL60 (17.5 μM), A549 (11.0 μM), SMMC-7721 (16.8 μM), MCF-7 (17.5 μM), and SW480 (18.0 μM)	Cytotoxic	[166]
*Euphorbia ebracteolata* Hayata	Roots	Ethanol	Yuexiandajisu F	HL60, A549, SMMC-7721, MCF-7, and SW480	HL60 (16.8 μM), A549 (19.7 μM), SMMC-7721 (18.4 μM), MCF-7 (15.3 μM), and SW480 (15.3 μM)	Cytotoxic	[166]
*Euphorbia ebracteolata* Hayata	Roots	Ethanol	jolkinol B	HL60, A549, SMMC-7721, MCF-7, and SW480	HL60 (5.0 μM), A549 (11.5 μM), SMMC-7721 (3.5 μM), MCF-7 (15.8 μM), and SW480 (9.5 μM)	Cytotoxic	[166]
*Euphorbia fischeriana* Steud	Roots	Ethanol	ebracteolatas D	HCT116, A549, HeLa, SW620, MCF-7, HepG-2	HCT116 (>40 μM), A549 (22.03 μM), HeLa (>40 μM), SW620 (>40 μM), MCF-7 (>40 μM), HepG-2 (>40 μM)	Cytotoxic	[48]
*Euphorbia fischeriana* Steud	Roots	Ethanol	ebractenoid Q	HCT116, A549, HeLa, SW620, MCF-7, HepG-2	HCT116 (33.18 μM), A549 (2.81 μM), HeLa (>40 μM), SW620 (>40 μM), MCF-7 (>40 μM), HepG-2 (27.24 μM)	Cytotoxic	[48]
*Euphorbia fischeriana* Steud	Roots	Ethanol	Euphonoid H	MDA-MB-231, HCT-15, RKO, C4-2B, C4-2B/ENZR	MDA-MB-231 (21.80 ± 2.35 μM), HCT-15 (28.57 ± 1.16 μM), RKO (20.46 ± 1.43 μM), C4-2B (5.52 ± 0.65 μM), C4-2B/ENZR (4.16 ± 0.42 μM)	Cytotoxic	[47]
*Euphorbia fischeriana* Steud	Roots	Ethanol	Euphonoid I	MDA-MB-231, HCT-15, RKO, C4-2B, C4-2B/ENZR	MDA-MB-231 (7.95 ± 0.82 μM), HCT-15 (12.45 ± 3.24 μM), RKO (8.78 ± 2.45 μM), C4-2B (4.49 ± 0.78 μM), C4-2B/ENZR (5.74 ± 0.45 μM)	Cytotoxic	[47]
*Euphorbia fischeriana* Steud	Roots	EtOH	17-Hydroxyljolkinolide B	MCF-10A, MCF-7, ZR-75-1 and MDA-MB-231	MCF-10A (3.4 ± 0.1 μg/mL), MCF-7 (4.7 ± 0.2 μg/mL), ZR-75-1 (2.2 ± 0.1 μg/mL) and MDA-MB-231 (1.1 ± 0.1 μg/mL)	Cytotoxic	[167]
*Euphorbia fischeriana* Steud	Roots	EtOH	17-Acetyljolkinolide B	MCF-10A, MCF-7, ZR-75-1 and MDA-MB-231	MCF-10A (4.3 ± 0.1 μg/mL), MCF-7 (3.4 ± 0.1 μg/mL), ZR-75-1 (1.2 ± 0.1 μg/mL) and MDA-MB-231 (1.7 ± 0.1 μg/mL)	Cytotoxic	[167]
*Euphorbia fischeriana* Steud	Roots	Ethanol	Euphorfiatnoid B	HepG2, H460, and MCF-7	H460 (9.97 μM)	Cytotoxic	[168]
*Euphorbia fischeriana* Steud	Roots	Ethanol	Euphorfiatnoid A	HepG2, H460, and MCF-7	HepG2 (11.64 μM), H460 (28.54 ± 1.20 μM), and MCF-7 (40.02 ± 0.47 μM)	Cytotoxic	[168]
*Euphorbia fischeriana* Steud	Roots	Ethanol	Euphorfiatnoid C	HepG2, H460, and MCF-7	HepG2 (13.10 ± 0.35 μM), H460 (14.88 ± 0.57 μM), and MCF-7 (32.95 ± 0.40 μM)	Cytotoxic	[168]
*Euphorbia fischeriana* Steud.	Roots	Ethanol	Euphorfischerin A	HeLa, N460 and Namalwa	HeLa (4.6 ± 0.11 μM), N460 (11.5 ± 0.04 μM) and Namalwa (16.4 ± 0.07 μM)	Cytotoxic	[169]
*Euphorbia fischeriana* Steud.	Roots	Ethanol	Euphorfischerin B	HeLa, N460 and Namalwa	HeLa (9.5 ± 0.16 μM), N460 (17.4 ± 0.34 μM) and Namalwa (13.3 ± 0.19 μM)	Cytotoxic	[169]
*Euphorbia fischeriana* Steud.	Aerial parts	Methanol	3α-acetoxy-14-hydroxy-*ent*-abieta-8(9),13(15)-dien-16,12-olide	HL-60, SMMC-7721 and SGC-7901	HL-60 (15.3 μM), SMMC-7721 (23.2 μM) and SGC-7901 (29.0 μM)	Cytotoxic	[170]
*Euphorbia helioscopia* L.	Aerial parts	Ethanol aqueous	Euphoheliphane A	OS-RC-2, Ketr-3, 769-P, G401, GRC-1 and ACHN	OS-RC-2 (47 μM), Ketr-3 (45 μM), 769-P (43 μM), G401 (38 μM), GRC-1 (41 μM) and ACHN (40 μM)	Cytotoxic	[171]
*Euphorbia helioscopia* L.	Aerial parts	Ethanol aqueous	Euphoheliphane B	OS-RC-2, Ketr-3, 769-P, G401, GRC-1 and ACHN	OS-RC-2 (31 μM), Ketr-3 (32 μM), 769-P (30 μM), G401 (34 μM), GRC-1 (33 μM) and ACHN (35 μM)	Cytotoxic	[171]
*Euphorbia helioscopia* L.	Aerial parts	Ethanol aqueous	Euphoheliphane C	OS-RC-2, Ketr-3, 769-P, G401, GRC-1 and ACHN	OS-RC-2 (35 μM), Ketr-3 (41 μM), 769-P (39 μM), G401 (32 μM), GRC-1 (38 μM) and ACHN (36 μM)	Cytotoxic	[171]
*Euphorbia heliosocpia* L	Whole plant	Methanol	Euphohelinoid A	HepG2, Hela, HL-60, SMMC-7721	HepG2 (24.3 ± 1.5 μM), Hela (28.4 ± 1.8 μM), HL-60 (18.6 ± 1.1 μM), SMMC-7721 (29.6 ± 1.5 μM)	Cytotoxic	[172]
*Euphorbia heliosocpia* L	Whole plant	Methanol	Euphohelinoid B	HepG2, Hela, HL-60, SMMC-7721	HepG2 (10.2 ± 1.4 μM), Hela (9.3 ± 1.2 μM), HL-60 (8.1 ± 0.7 μM), SMMC-7721 (9.8 ± 1.3 μM)	Cytotoxic	[172]
*Euphorbia heliosocpia* L	Whole plant	Methanol	Euphohelinoid C	HepG2, Hela, HL-60, SMMC-7721	HepG2 (>50 μM), Hela (>50 μM), HL-60 (>50 μM), SMMC-7721 (>50 μM)	Cytotoxic	[172]
*Euphorbia heliosocpia* L	Whole plant	Methanol	Euphohelinoid D	HepG2, Hela, HL-60, SMMC-7721	HepG2 (>50 μM), Hela (34.5 ± 2.3 μM), HL-60 (34.1 ± 1.6 μM), SMMC-7721 (30.1 ± 1.9 μM)	Cytotoxic	[172]
*Euphorbia heliosocpia* L	Whole plant	Methanol	Euphohelinoid F	HepG2, Hela, HL-60, SMMC-7721	HepG2 (12.5 ± 1.6 μM), Hela (14.1 ± 0.8 μM), HL-60 (13.3 ± 1.2 μM), SMMC-7721 (11.1 ± 1.7 μM)	Cytotoxic	[172]
*Euphorbia heliosocpia* L	Whole plant	Methanol	euphornin L	HepG2, Hela, HL-60, SMMC-7721	HepG2 (22.8 ± 1.7 μM), Hela (25.7 ± 2.2 μM), HL-60 (13.1 ± 1.8 μM), SMMC-7721 (14.3 ± 2.2 μM)	Cytotoxic	[172]
*Euphorbia heliosocpia* L	Whole plant	Methanol	helioscopianoid O	HepG2, Hela, HL-60, SMMC-7721	HepG2 (>50 μM), Hela (26.2 ± 1.4 μM), HL-60 (18.2 ± 1.9 μM), SMMC-7721 (19.5 ± 1.2 μM)	Cytotoxic	[172]
*Euphorbia heliosocpia* L	Whole plant	Methanol	euphoscopin I	HepG2, Hela, HL-60, SMMC-7721	HepG2 (24.1 ± 1.2 μM), Hela (29.7 ± 2.1 μM), HL-60 (14.3 ± 1.1 μM), SMMC-7721 (18.7 ± 1.1 μM)	Cytotoxic	[172]
*Euphorbia heliosocpia* L	Whole plant	Methanol	euphoscopin J	HepG2, Hela, HL-60, SMMC-7721	HepG2 (14.9 ± 1.3 μM), Hela (13.7 ± 1.4 μM), HL-60 (12.4 ± 1.2 μM), SMMC-7721 (15.0 ± 1.7 μM)	Cytotoxic	[172]
*Euphorbia heliosocpia* L	Whole plant	Methanol	euphoscopin B	HepG2, Hela, HL-60, SMMC-7721	HepG2 (23.3 ± 1.3 μM), Hela (29.2 ± 1.6 μM), HL-60 (20.2 ± 1.5 μM), SMMC-7721 (27.1 ± 1.4 μM)	Cytotoxic	[172]
*Euphorbia heterophylla* L.	Roots	*n*-hexane, DCM and MeOH	13-epicupressic acid	EJ-138, HepG2, A549, MCF-7 and PC3	EJ-138 (>200 μg/mL), HepG2 (>200 μg/mL), A549 (157.4 ± 3.24 μg/mL), MCF-7 (139.1 ± 2.14 μg/mL) and PC3 (>200 μg/mL)	Cytotoxic	[173]
*Euphorbia heterophylla* L.	Roots	*n*-hexane, DCM and MeOH	imbricatholic acid	EJ-138, HepG2, A549, MCF-7, and PC3	EJ-138 (>200 μg/mL), HepG2 (>200 μg/mL), A549 (>200 μg/mL), MCF-7 (173.5 ± 4.34 μg/mL) and PC3 (>200 μg/mL)	Cytotoxic	[173]
*Euphorbia heterophylla* L.	Roots	*n*-hexane, DCM and MeOH	α-hydroxy sandaracopimaric acid	EJ-138, HepG2, A549, MCF-7, and PC3	EJ-138 (173.3 ± 2.37 μg/mL), HepG2 (>200 μg/mL), A549 (>200 μg/mL), MCF-7 (>250 μg/mL) and PC3 (>250 μg/mL)	Cytotoxic	[173]
*Euphorbia heterophylla* L.	Roots	*n*-hexane, DCM and MeOH	13-epicupressic acid	EJ-138, HepG2, A549, MCF-7, and PC3	EJ-138 (182.2 ± 1.18 μg/mL), HepG2 (>200 μg/mL), A549 (>200 μg/mL), MCF-7 (>200 μg/mL) and PC3 (>250 μg/mL)	Cytotoxic	[173]
*Euphorbia heterophylla* L.	Roots	*n*-hexane, DCM and MeOH	β-hydroxy sandaracopimaric acid 13-epicupressic acid	EJ-138, HepG2, A549, MCF-7, and PC3	EJ-138 (>200 μg/mL), HepG2 (>200 μg/mL), A549 (67.4 ± 2.45 μg/mL), MCF-7 (111.7 ± 3.75 μg/mL) and PC3 (>200 μg/mL)	Cytotoxic	[173]
*Euphorbia heterophylla* L.	Roots	*n*-hexane, DCM and MeOH	5,7,3′,4′-pentahydroxyflavone	EJ-138, HepG2, A549, MCF-7, and PC3	EJ-138 (>200 μg/mL), HepG2 (>200 μg/mL), A549 (135.8 ± 7.41 μg/mL), MCF-7 (117.4 ± 3.71 μg/mL) and PC3 (>200 μg/mL)	Cytotoxic	[173]
*Euphorbia heterophylla* L.	Roots	*n*-hexane, DCM and MeOH	quercitrin	EJ-138, HepG2, A549, MCF-7, and PC3	EJ-138 (>250 μg/mL), HepG2 (>200 μg/mL), A549 (138.1 ± 4.62 μg/mL), MCF-7 (105.3 ± 6.19 μg/mL), and PC3 (>200 μg/mL)	Cytotoxic	[173]
*Euphorbia hypericifolia* L	Whole herb	Ethanol	euphypenoid A	HCT-116	HCT-116 (12.8 ± 1.6 μM)	Cytotoxic	[174]
*Euphorbia hypericifolia* L	Whole herb	Ethanol	20(S),24(R)-20,24-epoxy-24-methyldammaran-3β-ol	HCT-116	HCT-116 (26.8 ± 4.6 μM)	Cytotoxic	[174]
*Euphorbia hypericifolia* L	Whole herb	Ethanol	3β-hydroxycycloart-24-one	HCT-116	HCT-116 (7.4 ± 0.2 μM)	Cytotoxic	[174]
*Euphorbia hypericifolia* L	Whole herb	Ethanol	isomotiol	HCT-116	HCT-116 (10.6 ± 1.2 μM)	Cytotoxic	[174]
*Euphorbia kansui* S.L.Liou ex S.B.Ho	Roots	Ethanol	Euphorikanin A	HeLa and NCI-446	HeLa (28.85 ± 1.41 μM) and NCI-446 (20.89 ± 1.67 μM)	Cytotoxic	[175]
*Euphorbia lactea* Haw.	Aerial parts	Ethanol, *n*-hexane fraction	friedelin	HN22, HepG2, and HCT116	-	Cytotoxic	[149]
*Euphorbia lactea* Haw.	Aerial parts	Ethanol, *n*-hexane fraction	taraxerol	HN22, HepG2, and HCT116	-	Cytotoxic	[149]
*Euphorbia lactea* Haw.	Aerial parts	Ethanol, *n*-hexane fraction	friedelan-3α-ol	HN22, HepG2, and HCT116	-	Cytotoxic	[149]
*Euphorbia lagascae* Spreng.	Seeds	Methanol	Esculetin	LoVo and LoVo/Dx	LoVo (56.81 ± 5.42%) and LoVo/Dx (68.42 ± 7.56%)	Cytotoxic	[145]
*Euphorbia microsphaera* Boiss	Aerial parts	Hexane, chloroform and methanol	Aryanin (3aR,4S,4aS,5R,7aS,9aS)-5-hydroxy-5,8-dimethyl-3-methylene-2-oxo2,3,3a,4,4a,5,6,7,7a, 9a decahydroazuleno [6,5-b] furan-4-yl acetate)	MCF-7	MCF-7 (13.81 μg/mL)	Cytotoxic	[61]
*Euphorbia nematocypha* Hand.-Mazz.	Roots	Ethanol	16-O-caffeoyl-16-hydroxyldodecanoic acid	MCF-7 and HeLa	MCF-7 (20.22 ± 1.2 µmol/L) and HeLa (27.8 ± 1.4 µmol/L)	Cytotoxic	[176]
*Euphorbia nematocypha* Hand.-Mazz.	Aerial parts	Methylene chloride	*trans*, *trans*-2′,4′-hexadienedioicacid-1′-β-amyrin ester	MCF7 and HeLa	MCF7 (29.5 ± 3.4 μmol /L) and HeLa (23.2± 4.2 μmol /L)	Cytotoxic	[177]
*Euphorbia nematocypha* Hand.-Mazz.	Roots	Ethanol	Nematocynine	HCC1806, ST486, CT26, HeLa, and A549	HCC1806 (16.96 ± 0.16 μM), ST486 (60.94 ± 0.74 μM), CT26 (52.04 ± 1.96 μM), and HeLa (52.70 ± 0.52 μM)	Cytotoxic	[178]
*Euphorbia neriifolia* Linn.	Aerial parts	Ethanol	Phonerilin B	A549 and HL60	A549 (8.6 ± 1.7 μM) and HL60 (9.1 ± 0.02 μM)	Cytotoxic	[179]
*Euphorbia neriifolia* Linn.	Aerial parts	Ethanol	Phonerilin E	A549 and HL60	A549 (4.9 ± 0.06 μM) and HL60 (9.2 ± 0.09 μM)	Cytotoxic	[179]
*Euphorbia neriifolia* Linn.	Aerial parts	Ethanol	Phonerilin F	A549 and HL60	A549 (3.8 ± 0.2 μM) and HL60 (4.5 ± 0.7 μM)	Cytotoxic	[179]
*Euphorbia neriifolia* Linn.	Aerial parts	Ethanol	Phonerilin H	A549 and HL60	A549 (7.5 ± 0.8 μM) and HL60 (5.7 ± 1.0 μM)	Cytotoxic	[179]
*Euphorbia neriifolia* Linn.	Aerial parts	Ethanol	20-O-diacetyl-ingenol	A549 and HL60	HL60 (3.1 ± 0.2 μM)	Cytotoxic	[179]
*Euphorbia neriifolia* Linn.	Aerial parts	Ethanol	7,12-O-diacetyl-8-O-tigloylingol	A549 and HL60	A549 (6.4 ± 0.2 μM) and HL60 (9.5 ± 0.04 μM)	Cytotoxic	[179]
*Euphorbia osyridea* Bioss.	Aerial flowering parts	Dichloromethane/Acetone	2,7,9,14-tetraacetyl-3-benzoyl-8-butanoyl-5,15-dihydroxy-6(17),11(E)-jatrophadiene	Caov-4, and OVCAR	Caov-4 (46.27 ± 3.86 μM) and OVCAR (38.81 ± 3.30 μM)	Cytotoxic	[180]
*Euphorbia osyridea* Bioss.	Aerial flowering parts	Dichloromethane/Acetone	2,7,9,14-tetraacetyl-3-benzoyl-propionyl ester-5,15-dihydroxy-6(17),11(E)-jatrophadiene	Caov-4, and OVCAR	Caov-4 (36.48 ± 3.18), and OVCAR (42.59 ± 4.50 μM)	Cytotoxic	[180]
*Euphorbia pekinensis* Rupr.	Roots	Ethanol	Pekinenin G (11a,12b-epoxy-18-hydroxy-1bH, 2aH-casba-3E and 7E-dien-5-one)	BGC823, A549, HT-29, and MCF-7	BGC823 (42.7 μM), A549 (40.8 μM), HT-29 (47.8 μM), and MCF-7 (48.5 μM)	Cytotoxic	[181]
*Euphorbia pekinensis* Rupr.	Roots	Ethanol	Pekinenin G (11a,12b-epoxy-18-hydroxy-1bH, 2aH-casba-3E and 7E-dien-5-one) 2	BGC823, A549, HT-29, and MCF-8	BGC823 (53.9 μM), A549 (88.8 μM), HT-29 (70.1 μM), and MCF-7 (70.5 μM)	Cytotoxic	[181]
*Euphorbia pekinensis* Rupr.	Roots	Ethanol	Pekinenin G (11a,12b-epoxy-18-hydroxy-1bH, 2aH-casba-3E and 7E-dien-5-one) 3	BGC823, A549, HT-29, and MCF-9	BGC823 (15.6 μM), A549 (21.9 μM), HT-29 (25.1 μM), and MCF-7 (22.3 μM)	Cytotoxic	[181]
*Euphorbia pekinensis* Rupr.	Roots	Ethanol	Pekinenin G (11a,12b-epoxy-18-hydroxy-1bH, 2aH-casba-3E and 7E-dien-5-one) 4	BGC823, A549, HT-29, and MCF-10	BGC823 (25.1 μM), A549 (35.1 μM), HT-29 (30.2 μM), and MCF-7 (32.3 μM)	Cytotoxic	[181]
*Euphorbia pekinensis* Rupr.	Roots	Ethanol	Pekinenin G (11a,12b-epoxy-18-hydroxy-1bH, 2aH-casba-3E and 7E-dien-5-one) 5	BGC823, A549, HT-29, and MCF-11	BGC823 (54.8 μM), A549 (90.2 μM), HT-29 (110.7 μM), and MCF-7 (87.9 μM)	Cytotoxic	[181]
*Euphorbia pekinensis* Rupr.	Roots	Ethanol	Pekinenin G (11a,12b-epoxy-18-hydroxy-1bH, 2aH-casba-3E and 7E-dien-5-one) 6	BGC823, A549, HT-29, and MCF-12	BGC823 (12.1 μM), A549 (15.6 μM), HT-29 (11.3 μM), and MCF-7 (21.2 μM)	Cytotoxic	[181]
*Euphorbia pekinensis* Rupr.	Roots	Ethanol	(−)-(1S)-15-hydroxy-18-carboxycembrene	Hela, PC-3, HT1080, A375-S2, and MDA23	Hela (35.3 ± 3.6 μM), PC-3 (53.9 ± 6.2 μM), HT1080 (37.3 ± 2.0 μM), A375-S2 (28.7 ± 3.8 μM), and MDA24 (43.5 ± 5.1 μM)	Cytotoxic	[182]
*Euphorbia pekinensis* Rupr.	Roots	Ethanol	Jolkinol B	U-937 and LOVO	U-937 (3.60 ± 0.02 μM) and LOVO (8.44 ± 0.03 μM)	Cytotoxic	[183]
*Euphorbia pekinensis* Rupr.	Roots	Ethanol	Euphodane A	U-937	U-937 (5.92 ± 0.38 μM)	Cytotoxic	[183]
*Euphorbia pekinensis* Rupr.	Roots	Ethanol	Isopimara-7,15-dien-3β-ol	K-562	K-562 (0.87 ± 0.02 μM)	Cytotoxic	[183]
*Euphorbia pseudocactus* A.Berger	Aerial parts	Methanol	Gallic acid	LS-174T	LS-174T (18.27 μg/mL)	Cytotoxic	[184]
*Euphorbia pseudocactus* A.Berger	Aerial parts	Methanol	Ethyl gallate	LS-174T	LS-174T (25.42 μg/mL)	Cytotoxic	[184]
*Euphorbia royleana* Boiss.	Whole plant	Ethanol	(3b,23Z)-9,19-cyclolanost-23-ene-3,25-diol	A549	A549 (4.84 ± 0.56 μM)	Cytotoxic	[185]
*Euphorbia royleana* Boiss.	Whole plant	Ethanol	taraxerol	A549	A549 (7.11 ± 1.65 μM)	Cytotoxic	[185]
*Euphorbia sanctae-catharinae* Fayed	Aerial parts	Dichloromethane/Methanol (1:1)	4,12,20-trideoxyphorbol-13-(2,3-dimethyl) butyrate	A549 and Caco-2	A549 (3.3 (0.996) μM) and Caco-2 (29.4 (0.972) μM)	Cytotoxic	[175]
*Euphorbia schimperi* C.Presl	Aerial parts	MeOH/H_2_O (70:30 *V*/*V*)	Cycloschimperol A	MCF-7, HepG2, and HCT-116	MCF-7 (55.4 ± 3 μM), HepG2 (19.7 ± 3 μM), and HCT-116 (20.25 ± 5 μM)	Cytotoxic	[186]
*Euphorbia schimperi* C.Presl	Aerial parts	MeOH/H_2_O (70:30 *V*/*V*)	Cycloschimperol B	MCF-7, HepG2, and HCT-116	MCF-7 (2.1 ± 0.01 μM), HepG2 (1.4 ± 0.1 μM), and HCT-116 (1.8 ± 0.1 μM)	Cytotoxic	[186]
*Euphorbia schimperi* C.Presl	Aerial parts	MeOH/H_2_O (70:30 *V*/*V*)	Cycloart-25-en-3-one	MCF-7, HepG2, and HCT-116	MCF-7 (4.7 ± 0.1 μM), HepG2 (2.3 ± 0.2 μM), and HCT-116 (1.9 ± 0.4 μM)	Cytotoxic	[186]
*Euphorbia schimperiana* Scheele	Aerial parts	Ethanol	3,30-di-O-methylellagic acid	PC3	PC3 (5.5 mg/mL)	Cytotoxic	[187]
*Euphorbia sogdiana* Popov	Aerial parts	Acetone/Dichloromethane (1:2)	Tigliane diterpene	EJ-138 and Jurkat T	EJ-138 (12.1 μM) and Jurkat T (16.1 μM)	Cytotoxic	[188]
*Euphorbia stracheyi* Boiss	Whole plant	Methanol	3-O-benzoyl-20-deoxymgenol	HL-60, A-549, SMMC-7721, MCF-7, and SW480	HL-60 (0.5 ± 0.18 μM), A-549 (21.47 ± 0.17 μM), SMMC-7721 (18.36 ± 1.17 μM), MCF-7 (18.82 ± 0.84 μM), and SW481 (16.25 ± 0.71 μM)	Cytotoxic	[189]
*Euphorbia stracheyi* Boiss.	Roots	Methanol	Euphstrachenol A	MV4-11	MV4-11 (12.29 μM)	Cytotoxic	[190]
*Euphorbia stracheyi* Boiss.	Roots	Methanol	Euphstrachenol B	MV4-11	MV4-11 (14.80 μM)	Cytotoxic	[190]
*Euphorbia stracheyi* Boiss.	Roots	Methanol	Euphstrachenol C	MV4-11	MV4-11 (5.92 μM)	Cytotoxic	[190]
*Euphorbia taurinensis* All.	Whole plant	MeOH	Ingenane diterpene	L5178Y mouse T-lymphoma cells parent and MDR L5178Y	L5178Y mouse T-lymphoma cells parent (82.47 μM) and MDR L5178Y (62.81 μM)	Cytotoxic	[191]
*Euphorbia tirucalli* L.	Sap	Hexane	euphol	73 human cancer lines from 15 tumor types	Range from 1.41 to 38.89 μM	Cytotoxic	[192]
*Euphorbia tithymaloides* L.	Stems	Methanol	friedelane-3β-ol, 3-oxo-friedelane, euphane-7, 24-diene, 3β-ol (butyrospermol), and euphane -7, 25-diene, 3, 24-β- diols in addition to the diterpene derivative 1 α, 13 β, 14 α-trihydroxy-3 β, 7 β-dibenzenzoyloxy-9 β, 15 β-diacetoxyjatropha-5, 11-E-diene and the phytosterol β-sitosterol	HepG2, HCT-116, MCF-7 and PC-3	Only for compound: 1 α, 13 β, 14 α-trihydroxy-3 β, 7 β-dibenzenzoyloxy-9 β, 15 β-diacetoxyjatropha-5, 11-E-diene, HepG2(12.99 ± 0.9 μM), HCT-116(18.63 ± 1.4 μM), MCF-7 (24.40 ± 1.9 μM), and PC-3(37.12 ± 2.3 μM)	Cytotoxic	[164]
*Euphorbia umbellata* (Pax) Bruyns	Stems and leaves	Methanol	3,4,12,13-tetraacetylphorbol-20-phenylacetate	U251, MCF-7, NCI-ADR/RES, 786-0, NCI-H460, HT29, and K562	U251 (25.2 mg/mL), MCF-7 (>250 mg/mL), NCI-ADR/RES (>250 mg/mL), 786-0 (24.1 mg/mL), NCI-H460 (31.1 mg/mL), HT29 (>250 mg/mL), and K563 (65.3 mg/mL)	Cytotoxic	[127]
*Excoecaria agallocha* L.	Leaves and twigs	Ethanol	excagallonoid A	RKO	RKO (8.7 ± 1.98 μM)	Cytotoxic	[193]
*Excoecaria agallocha* L.	Leaves and twigs	Ethanol	2-hydroxy-atis-1,16-diene-3,14-dione	RKO	RKO (2.6 ± 2.81 μM)	Cytotoxic	[193]
*Jatropha gossypifolia* L.	Stem bark	-	Jatrophone	HepG2, WiDr, HeLa, and AGS	HepG2 (3.2 µM), WiDr (8.97 µM), HeLa (5.13), and AGS (2.5 µM)	Cytotoxic	[194]
*Jatropha gossypiifolia* L.	Branches and leaves	Ethanol	Jatrogrossidione	RKO	2.6 μM	Cytotoxicity Apoptosis associated with G2/M-phase cell cycle arrest.	[58]
*Jatropha tanjorensis* J.L.Ellis & Saroja	Leaves	Hexane, chloroform and methanol	R (+) 4-hydroxy-2-pyrrolidinone	HEP-2, B16F10, A549, and NRK 49F	HEP-2 (42.26 ± 0.03 μg/mL), B16F10 (44.56 ± 0.02 μg/mL), A549 (48.26 ± 0.03 μg/mL), and NRK 49F (47.28 ± 0.03 μg/mL).	Cytotoxic	[195]
*Macaranga barteri* Müll.Arg.	Leaves	*N*-hexane, dichloromethane and methanol	macabartebenes A	MCF7, HeLa, A549, and PC3	MCF7 (0.68 ± 0.01 μM), HeLa (0.60 ± 0.01 μM), A549 (0.79 ± 0.01 μM), and PC3 (0.66 ± 0.01 μM)	Cytotoxic	[196]
*Macaranga barteri* Müll.Arg.	Leaves	*N*-hexane, dichloromethane and methanol	macabartebenes B	MCF7, HeLa, A549, and PC3	MCF7 (0.71 ± 0.02 μM), HeLa (0.72 ± 0.01 μM), A549 (0.74 ± 0.01 μM), and PC3 (0.69 ± 0.01 μM)	Cytotoxic	[196]
*Macaranga barteri* Müll.Arg.	Leaves	*N*-hexane, dichloromethane and methanol	macabartebenes C	MCF7, HeLa, A549, and PC3	MCF7 (1.73 ± 0.01 μM), HeLa (1.67 ± 0.01 μM), A549 (1.81 ± 0.00 μM), and PC3 (1.61 ± 0.01 μM)	Cytotoxic	[196]
*Macaranga barteri* Müll.Arg.	Leaves	*N*-hexane, dichloromethane and methanol	vedelianin	MCF7, HeLa, A549, and PC3	MCF7 (0.32 ± 0.03 μM), HeLa (0.51 ± 0.01 μM), A549 (0.54 ± 0.02 μM), and PC3 (0.39 ± 0.01 μM)	Cytotoxic	[196]
*Macaranga barteri* Müll.Arg.	Leaves	*N*-hexane, dichloromethane and methanol	schweinfurthin G	MCF7, HeLa, A549, and PC3	MCF7 (0.95 ± 0.02 μM), HeLa (1.18 ± 0.01 μM), A549 (1.10 ± 0.09 μM), and PC3 (0.91 ± 0.01 μM)	Cytotoxic	[196]
*Macaranga barteri* Müll.Arg.	Leaves	*N*-hexane, dichloromethane and methanol	8-prenylkaempferol	MCF7, HeLa, A549, and PC3	MCF7 (6.22 ± 0.13 μM), HeLa (6.88 ± 0.16 μM), A549 (6.61 ± 0.21 μM), and PC3 (6.53 ± 0.11 μM)	Cytotoxic	[196]
*Macaranga barteri* Müll.Arg.	Leaves	*N*-hexane, dichloromethane and methanol	mappain	MCF7, HeLa, A549, and PC3	MCF7 (0.71 ± 0.02 μM), HeLa (0.71 ± 0.01 μM), A549 (0.81 ± 0.02 μM), and PC3 (0.77 ± 0.01 μM)	Cytotoxic	[196]
*Macaranga barteri* Müll.Arg.	Leaves	*N*-hexane, dichloromethane and methanol	broussoflavonol F	MCF7, HeLa, A549, and PC3	MCF7 (4.13 ± 0.00 μM), HeLa (4.10 ± 0.01 μM), A549 (3.83 ± 0.01 μM), and PC3 (3.99 ± 0.01 μM)	Cytotoxic	[196]
*Macaranga barteri* Müll.Arg.	Leaves	*N*-hexane, dichloromethane and methanol	isomacarangin	MCF7, HeLa, A549, and PC3	MCF7 (8.43 ± 0.26 μM), HeLa (8.49 ± 0.21 μM), A549 (8.72 ± 0.21 μM), and PC3 (8.5 ± 0.31 μM)	Cytotoxic	[196]
*Macaranga gigantea* (Rchb.f. & Zoll.) Müll.Arg.	Leaves	Methanol	Glyasperin	P-388	P-388 (3.44 μg/mL)	Cytotoxic	[197]
*Macaranga gigantea* (Rchb.f. & Zoll.) Müll.Arg.	Leaves	Methanol	Meliternatin	P-388	P-388 (30.04 μg/mL)	Cytotoxic	[197]
*Macaranga gigantifolia* Merr.	Leaves	Methanol, ethyl acetate fraction	Apigenin	P-388	P-388 (14.13 μg/mL)	Cytotoxic	[64]
*Macaranga hispida* (Blume) Mull.Arg	Leaves	Methanol	5,7,3′,4′-tetrahydroxy-6-geranyl flavonol	P388	P388 (0.22 μg/mL)	Cytotoxic	[19]
*Macaranga hispida* (Blume) Mull.Arg	Leaves	Methanol	kaemferol 7–O-β-glucoside	P388	P388 (101.5 μg/mL)	Cytotoxic	[19]
*Macaranga kurzii* (Kuntze) Pax & K.Hoffm.	Twigs	Ethanol	kurzphenol A	HepG2	HepG2 (30.14 μg/mL)	Cytotoxic	[198]
*Macaranga kurzii* (Kuntze) Pax & K.Hoffm.	Twigs	Ethanol	kurzphenol C	A549	A549 (17.11 μg/mL)	Cytotoxic	[198]
*Macaranga kurzii* (Kuntze) Pax & K.Hoffm.	Twigs	Ethanol	8-prenylnaringenin	A549	A549 (9.76 μg/mL)	Cytotoxic	[198]
*Macaranga kurzii* (Kuntze) Pax & K.Hoffm.	Twigs	Ethanol	glepidotin B	A549	A549 (15.32 μg/mL)	Cytotoxic	[198]
*Macaranga kurzii* (Kuntze) Pax & K.Hoffm.	Twigs	Ethanol	acetylatractylodinol	A549	A549 (18.22 μg/mL)	Cytotoxic	[198]
*Macaranga kurzii* (Kuntze) Pax & K.Hoffm.	Twigs	Ethanol	blumenol A	A549	A549 (18.23 μg/mL)	Cytotoxic	[198]
*Macaranga kurzii* (Kuntze) Pax & K.Hoffm.	Twigs	Ethanol	alicylic acid	A549	A549 (12.01 μg/mL)	Cytotoxic	[198]
*Macaranga recurvata* Gage	Leaves	Methanol	Macarecurvatin A	MCF7 and MCF7/TAMR	MCF7 (5.26 μM) and MCF7/TAMR (5.66 μM)	Cytotoxic	[199]
*Macaranga recurvata* Gage	Leaves	Methanol	Macarecurvatin B	MCF7 and MCF7/TAMR	MCF7 (0.96 μM) and MCF7/TAMR (1.25 μM)	Cytotoxic	[199]
*Macaranga recurvata* Gage	Leaves	Methanol	6,8-diisoprenylaromadendrin	MCF7 and MCF7/TAMR	MCF7 (5.03 μM) and MCF7/TAMR (5.83 μM)	Cytotoxic	[199]
*Mallotus conspurcatus* Croizat	Aerial parts	Methanol	6-Prenylnaringenin	HeLa and A549	HeLa (30.12 ± 1.21 μM) and A549 (70.25 ± 0.89 μM)	Cytotoxic	[200]
*Mallotus conspurcatus* Croizat	Aerial parts	Methanol	8-Prenylnaringenin	HeLa and A549	HeLa (60.16 ± 0.91 μM) and A549 (99.36 ± 1.94 μM)	Cytotoxic	[200]
*Mallotus conspurcatus* Croizat	Aerial parts	Methanol	7-O-Methyl-8-prenylnaringenin	HeLa and A549	HeLa (45.03 ± 0.82 μM) and A549 (89.16 ± 0.61 μM)	Cytotoxic	[200]
*Mallotus conspurcatus* Croizat	Aerial parts	Methanol	7-O-Methyl-6-prenylnaringenin	HeLa and A549	HeLa (19.69 ± 0.65 μM) and A549 (55.26 ± 1.87 μM)	Cytotoxic	[200]
*Mallotus conspurcatus* Croizat	Aerial parts	Methanol	4′-O-Methyl-6-prenylnaringenin	HeLa and A549	HeLa (10.08 ± 1.06 μM) and A549 (47.26 ± 0.82 μM)	Cytotoxic	[200]
*Manniophyton fulvum* Müll.Arg.	Twigs	Methanol	Betulinic acid	HeLa	4% at 62.5 μg/mL	Cytotoxic	[201]
*Mareya micrantha* Müll. Arg.	Leaves	Ethanol/Water	29-nor-2β,15α,20β-trihydroxy-16α-acetyl-3,1,22-trioxo-cucurbita-4,23-diene	Hep3B	Hep3B (0.12 ± 0.05 μM)	Cytotoxic	[202]
*Mareya micrantha* Müll. Arg.	Leaves	Ethanol/Water	29-nor-2β,15α,20β-trihydroxy-16α-acetyl-3,1,22-trioxo-cucurbita-4,23-diene 29-nor-1,2,3,4,5,10-dehydro-3,15α,20β-trihydroxy-16α-acetyl-11,22-dioxo-cucurbita-23-ene 2-O-β-D-glucopyranoside	Hep3B	Hep3B (43.8 ± 5.7 μM)	Cytotoxic	[202]
*Mareya micrantha* Müll. Arg.	Leaves	Ethanol/Water	29-nor-2β,15α,20β-trihydroxy-16α-acetyl-3,11,22 trioxo-cucurbita-4,23-diene 2-O-β-D glucopyranoside	Hep3B	Hep3B (>50 μM)	Cytotoxic	[202]
*Mareya micrantha* Müll. Arg.	Leaves	Ethanol/Water	dihydro-epi-isocucurbitacin D	Hep3B	Hep3B (18.2 ± 2.8 μM)	Cytotoxic	[202]
*Mareya micrantha* Müll. Arg.	Leaves	Ethanol/Water	tetrahydro-cucurbitacin I	Hep3B	Hep3B (14.9 ± 3.3 μM)	Cytotoxic	[202]
*Mareya micrantha* Müll. Arg.	Leaves	Ethanol/Water	cucurbitacin L	Hep3B	Hep3B (11.3 ± 6.2 μM)	Cytotoxic	[202]
*Margaritaria discoidea* (Baill.) G. L. Webste	Stem bark	Dichloromethane and methanol (1:1).	Securinine	OVCAR-8, A2780, and A2780cis	OVCAR-8 (16.2 ± 0.5 μM), A2780 (2.7 ± 0.7 μM), and A2780cis (6.5 ± 0.4 μM)	Cytotoxic	[203]
*Margaritaria discoidea* (Baill.) G. L. Webste	Stem bark	Dichloromethane and methanol (1:1).	Gallic acid	OVCAR-8, A2780, and A2780cis	OVCAR-8 (5.2 ± 0.1 μM), A2780 (6.2 ± 0.3 μM), and A2780cis (5.4 ± 0.3 μM)	Cytotoxic	[203]
*Ricinodendron heudelotii* (Baill.) Heckel	Leaves	Ethanol	Corilagin	HL-60, SMMC-7721, A-549, MCF-7, and SW-480	HL-60 (25.81 ± 0.67 μg/mL), MCF-7 (33.18 ± 0.76 μg/mL), and SW-480 (37.04 ± 1.06 μg/mL)	Cytotoxic	[204]
*Suregada zanzibariensis* Baill.	Stem bark	Dichloromethane/Methanol (1:1)	Mangiolide	TK10, UACC62, and MCF7	TK10 (0.02 μg/mL), UACC62 (0.03 μg/mL), and MCF7 (0.05 μg/mL)	Cytotoxic	[205]
*Suregada zanzibariensis* Baill.	Stem bark	Dichloromethane/Methanol (1:1)	Jolkinolide B	TK10, UACC62, and MCF8	TK10 (3.31 μg/mL), UACC62 (0.94 μg/mL), and MCF7 (2.99 μg/mL)	Cytotoxic	[205]
*Trewia nudiflora* L.	Fruits	Ethanol	*N*-methyltreflorine	HeLa, MV-4–11, MCF-7, and MCF-7/ADR	HeLa (0.54 nM), MV-4–11 (3.6 nM), MCF-7 (8.6 nM), and MCF-7/ADR (13 nM)	Cytotoxic and in- hibited tubulin polymerization in vitro	[206]
*Trewia nudiflora* L.	Fruits	Ethanol	Methyltrewiasine	HeLa, MV-4–11, MCF-7, and MCF-7/ADR	HeLa (1.6 nM), MV-4–11 (3.1 nM), and MCF-7 (10 nM)	Cytotoxic and in- hibited tubulin polymerization in vitro	[206]
*Trewia nudiflora* L.	Fruits	Ethanol	Treflorine	HeLa, MV-4–11, MCF-7, and MCF-7/ADR	HeLa (0.74 nM), MV-4–11 (0.12 nM), and MCF-7 (5.5 nM)	Cytotoxic	[206]
*Trewia nudiflora* L.	Fruits	Ethanol	Trenudine	HeLa, MV-4–11, MCF-7, and MCF-7/ADR	HeLa (0.41 nM), MV-4–11 (4.8 nM), MCF-7 (11 nM), and MCF-7/ADR (28)	Cytotoxic	[206]
*Trewia nudiflora* L.	Fruits	Ethanol	Colubrinol	HeLa, MV-4–11, MCF-7, and MCF-7/ADR	HeLa (0.28 nM), MV-4–11 (0.21 nM), and MCF-7 (3.2 nM)	Cytotoxic	[206]
*Trigonostemon heterophyllus* Merr.	Stems and leaves	Ethanol	Trigoheterophines A	HL60, SMMC-7721, A-549, MCF-7, and SW480	HL60 (0.58 ± 0.06 μM), SMMC-7721 (1.42 ± 0.07 μM), A-549 (3.18 ± 0.11 μM), MCF-7 (0.28 ± 0.02 μM), and SW480 (0.93 ± 0.05 μM)	Antiproliferative	[207]
*Trigonostemon heterophyllus* Merr.	Stems and leaves	Ethanol	Trigoheterophines B	HL60, SMMC-7721, A-549, MCF-7, and SW480	HL60 (0.66 ± 0.04 μM), SMMC-7721 (1.98 ± 0.08 μM), A-549 (0.52 ± 0.03 μM), MCF-7 (0.75 ± 0.05 μM), and SW480 (2.08 ± 0.11 μM)	Antiproliferative	[207]
*Trigonostemon heterophyllus* Merr.	Stems and leaves	Ethanol	(Z, Z, E, E)-1, 4-epoxy-16-hydroxyheneicos-1, 3, 12, 14- tetraene	HL60, SMMC-7721, A-549, MCF-7, and SW480	HL60 (2.12 ± 0.10 μM), SMMC-7721 (3.26 ± 0.09 μM), A-549 (2.20 ± 0.07 μM), MCF-7 (1.68 ± 0.06 μM), and SW480 (2.72 ± 0.08 μM)	Antiproliferative	[207]
*Trigonostemon heterophyllus* Merr.	Stems and leaves	Ethanol	(Z, Z, E, E, E)-1, 4-epoxy-16-hydroxyheneicos-1, 3, 12, 14, 18-pentaene	HL60, SMMC-7721, A-549, MCF-7, and SW480	HL60 (3.98 ± 0.12 μM), SMMC-7721 (1.42 ± 0.07 μM), A-549 (3.18 ± 0.11 μM), MCF-7 (0.45 ± 0.05 μM), and SW480 (2.23 ± 0.10 μM)	Antiproliferative	[207]
*Trigonostemon heterophyllus* Merr.	Stems and leaves	Ethanol	2-(hexadecyl)furan	HL60, SMMC-7721, A-549, MCF-7, and SW480	HL60 (2.07 ± 0.06 μM), SMMC-7721 (1.83 ± 0.03 μM), A-549 (4.86 ± 0.10 μM), MCF-7 (1.78 ± 0.06 μM), and SW480 (4.28 ± 0.09 μM)	Antiproliferative	[207]
*Trigonostemon heterophyllus* Merr.	Stems and leaves	Ethanol	2-(octadecyl)furan	HL60, SMMC-7721, A-549, MCF-7, and SW480	HL60 (1.05 ± 0.06 μM), SMMC-7721 (2.97 ± 0.13 μM), A-549 (6.32 ± 0.15 μM), MCF-7 (3.02 ± 0.07 μM), and SW480 (12.06 ± 0.11 μM)	Antiproliferative	[207]
*Trigonostemon xyphophylloides* (Croizat) L.K.Dai & T.L.Wu	Twigs	Ethanol	Trigoxyphin P	SPC-A-1, BEL-7402, SGC-7901, and K-562	SPC-A-1 (1.70 μM) and K-562 (2.24 μM)	Cytotoxic	[208]
*Trigonostemon xyphophylloides* (Croizat) L.K.Dai & T.L.Wu	Twigs	Ethanol	Trigoxyphin Q	SPC-A-1, BEL-7402, SGC-7901, and K-562	SPC-A-1 (1.42 μM), SGC-7901 (2.88 μM), and K-562 (0.37 μM)	Cytotoxic	[208]
*Trigonostemon xyphophylloides* (Croizat) L.K.Dai & T.L.Wu	Twigs	Ethanol	Trigoxyphin R	SPC-A-1, BEL-7402, SGC-7901, and K-562	SPC-A-1 (12.42 μM) and K-562 (17.18 μM)	Cytotoxic	[208]
*Trigonostemon xyphophylloides* (Croizat) L.K.Dai & T.L.Wu	Twigs	Ethanol	Trigoxyphin T	SPC-A-1, BEL-7402, SGC-7901, and K-562	SPC-A-1 (0.24 μM), BEL-7402 (3.89 μM), and SGC-7901 (5.59 μM)	Cytotoxic	[208]

**Table 3:** Cytotoxic properties of selected extracts or pure compounds from the Euphorbiaceae family against in vivo models.

Name of the Species	Part of the Plant	Type of Extract	Class of Compounds/Compounds Identified in Extract	Animal Model	Treatment	Activity/Mechanism/ Effect	Ref.
*Cnidosculos quercifolius* Pohl	Root bark	Methanol, chloroform fraction (Favelin rich fraction)	Favelin, Methyl-faveline, Deoxofavelin, Neofavelanone and Coumarin	Mice	250 and 500 mg/kg/day of favelin rich fraction.	Inhibition rates of tumor growth were 58.08 and 48.71% for the 250 mg/kg and 500 mg/kg treatment groups, respectively.	[212]
*Croton crassifolius* Geiseler	Roots	Ethanol	Penduliflaworosin	Mice and Rats	12.5–50 µM	Exerts its anti-angiogenic effect via the VEGF receptor-2 signaling pathway	[213]
*Euphorbia fischeriana* S. + *Ziziphus jujuba* M.	-	Water	Jokinolide B and 2,4-dihydroxy-6-methoxy-acetophenone	Mice	2.5, 5.0, and 10.0 g/kg groups of ESZM extract	PI3k/Akt pathway regulation of apoptosis.	[214]
*Euphorbia helioscopia* L.	Whole plant	Ethyl acetate	-	Mice	50 µg/mL, 100 µg/mL, and 200 µg/mL	Growth inhibition and Cyclin D1 protein expression decreased. Cell apoptosis by changing Bcl-2, Bax, and caspase-3 protein expressions.	[215]
*Euphorbia royleana* Boiss.	-	Hexane	-	Mice	10 mg/kg.	Tumor volumes were decreased. Necrotic areas in tumor tissue.	[216]
*Excoecaria agallocha* L.	Bark	Methanol	Quercetin-3-O-rutinoside, Quercetin 3-O- α -L-rhamnoside, Kaempferol-3-O-(2-O-acetyl-α-Lrhamnopyranoside), Kaempferol 3-O-α-Lrhamnopyranoside, Excoecarin A, Excoecarin G1, Excoecarin G2, Taraxerone, 3beta-[(2E,4E)-6-oxo-decadienoyloxy]- olean-12-ene, 2,3-secoatisane, Exoecarin B, Exoecarin C, Exoecarin D, Excoecarin E, Exoecarin F, Exoecarin H	Mice	Different doses up to 200 mg/kg	Normal hemaetological values.	[217]
*Tragia involucrata* L.	Whole plant	Ethanol	Phenylacetaldehyde- diethylacetal, Neophytadiene, (E)-Phytol, Ethyl palmitate, Phytol, Ethyl linolate, Ethyl elaidate, Linolenic acid, Ethyl octadecanoate, 2-Ethylhexyl phthalate, Squalene, Vitamin E, Clionasterol, Viminalol, Agathisflavone, Loquatoside, Leufolin A, Quercetin, Echinacin, Apigetrin, Cynaroside, 1,2,36-tetrakis-O- galloyl-B-D-glucose, Isoquercetin and Corilagin	Mice	200 mg/kg and 400 mg/kg	Reduction of tumors	[144]

Data not reported are represented by “-“.

**Table 4:** Nanoparticles made using constituents from the plants of the Euphobiaceae family.

Tested Plant	Components	Type of Nanoparticles	Cells	Effect	Ref.
*Acalypha wilkesiana* Müll.Arg.	Flowers	Ag NPs	MCF-7 (4.00 μg/mL) and PC-3 (3.60 μg/mL)	Cytotoxicity	[223]
*Alchornea cordifolia (Schumach. & Thonn.)* Müll.Arg.	Leaves	CuO–ZnO, ZnO, and CuO NPs	HeLa treatment with 100 μg/mL—CuO–ZnO (39.94 ± 5.01). ZnO (44.05 ± 0.91) and CuO NPs (63.64 ± 8.34)	Cytotoxicity	[224]
*Baliospermum montanum* (Willd.) Müll.Arg.	Roots	Nanoparticles	Aqueous NPS (22%) and Ethanol NPs (6%)	Cytotoxicity	[225]
*Croton sparsiflorus* Morong	Leaves	AuNPs	HepG2 (116.7 μg/mL)	Cytotoxicity	[226]
*Euphorbia dendroides* L.	Aerial parts	AuNPs	HepG2 (41.72 ± 1.26 mg/mL) and HCT-116 (44.96 ± 3.23 mg/mL)	Cytotoxicity	[227]
*Euphorbia heterophylla* L.	Leaves	rGO	A549 (297.81 mg/mL) and HepG2 (357.53 mg/mL)	Cytotoxicity	[228]
*Euphorbia peplus* L.	Leaves	AuNPs	HepG2 and Hela cells	Inhibitory effect	[229]
*Euphorbia royleana* Boiss.	Pulp	Ag NPs and Cu_2_O NPs	HCT-116 Ag NPs (50.12 μg/mL) and Cu_2_O NPs (61.93 μg/mL)	Cytotoxicity	[230]
*Excoecaria agallocha* L.	Leaves	AgNPs	1.00 lg/mL AgNPs in MCF-7 (8.00% viability)	Cytotoxicity	[231]

## Abbreviations

AgONPs	Silver oxide nanoparticles
Akt	Protein kinase B
APAF	Apoptotic Protease-Activating Factor-1
AuNPs	Gold nanoparticles
Bax	Apoptosis regulator or bcl-2-like protein 4
Bcl-2	B-cell lymphoma 2 protein
Casp-3	Caspase 3
Casp-6	Caspase 6
Casp-7	Caspase 7
Casp-8	Caspase 8
Casp-9	Caspase 9
CuONPs	Cupper oxide nanoparticles
MAPK	Mitogen-activated protein kinase
MMP-2	Metalloproteinase 2
MMP-9	Metalloproteinase 9
NP	Nanoparticles
P53	Tumor protein P53
PARP	Poly ADP-Ribose Polymerase
PI3K	Phosphoinositide 3-kinases
ROS	Reactive oxygen species
SMAC/DIABLO	Second Mitochondria-Derived Activator of Caspase
STAT3	Signal transducer and activator of transcription 3
TNBC	Triple negative breast cancer
TNB-Z	Tonantzitlolone B
XIAP	X-linked inhibitor of apoptosis
ZnONPs	Zinc oxide nanoparticles
